# Risk factors for omphalitis in neonatal dairy calves

**DOI:** 10.3389/fvets.2024.1480851

**Published:** 2024-11-25

**Authors:** Kim K. Meier, Annegret Stock, Roswitha Merle, Heidi Arndt, Linda Dachrodt, Martina Hoedemaker, Laura Kellermann, Gabriela Knubben-Schweizer, Maria Volkmann, Kerstin-Elisabeth Müller

**Affiliations:** ^1^Division for Ruminants and Camelids, Unit for Internal Medicine and Surgery, Department of Veterinary Medicine, Farm Animal Clinic, Freie Universität Berlin, Berlin, Germany; ^2^Department of Veterinary Medicine, Institute of Veterinary Epidemiology and Biostatistics, Freie Universität Berlin, Berlin, Germany; ^3^Clinic for Cattle, University of Veterinary Medicine Hannover, Foundation, Hannover, Germany; ^4^Department of Behavioral Physiology of Livestock, Institute of Animal Science, University of Hohenheim, Stuttgart, Germany; ^5^Clinic for Ruminants with Ambulatory and Herd Health Services, Centre for Clinical Veterinary Medicine, Ludwig-Maximilians-Universität Munich, Oberschleissheim, Germany

**Keywords:** omphalitis, risk factors, dairy calves, calving management, calf rearing, neonate

## Abstract

Knowledge about potential risk factors for animal health is crucial to achieve animal welfare. The aim of this study was to provide practical guidance for farmers to improve the health status of their youngstock by identifying and eliminating risk factors for omphalitis in neonatal calves. A cross-sectional study including 3,445 dairy calves from 567 farms located in three structurally different regions of Germany was performed from December 2016 to July 2019. On each farm calves aged five to 21 days underwent clinical examinations with special consideration of the umbilicus for signs of inflammation. Information regarding animal health, hygiene, and management was obtained via interviews with the farmers. Rearing conditions were recorded following visual inspection using written standard operating procedures. Multifactorial statistical analyses were performed to identify potential risk factors for omphalitis on animal and farm level. The overall omphalitis prevalence in calves aged five to 21 days was 30.9%. Across all regions and farms, every fourth calf per farm exhibited signs of omphalitis (median farm prevalence: 25.0%, interquartile range: 0.0–50.0%). According to the farmers, however, only 4.5% of the calves had been treated for omphalitis in the preceding 12 months. Risk factors for omphalitis identified included the dampness of the lying area in the first 2 weeks of life, a body condition score of the dam after calving outside the optimal range, and the time that calves spent with their dam after birth. Calves on farms providing dry lying areas in the first 2 weeks of life had 0.77 times the risk of omphalitis compared to calves on farms with predominantly damp bedding. When the dams were judged under- or over-conditioned after calving, their offspring had a 1.4 times higher omphalitis risk, respectively, compared to calves from dams optimal conditioned. Calves from farms separating calf and dam beyond 12 h after birth were 0.75 times as likely to develop omphalitis than calves from farms performing immediate separation. These results highlight the complexity of multifactorial diseases such as umbilical infection. The evidence presented can help to establish guidelines for dairy farmers to improve the umbilical health of their calves.

## 1 Introduction

Omphalitis is an infection of the umbilicus and its surrounding tissues, occurring primarily in the neonatal calf. In humans, it is considered a true medical emergency that can rapidly progress to systemic infection and death (1).

For dairy calves, the prevalence of omphalitis diagnosed by clinical examination of the individual animal has been reported to range from 3.8 to 28.7% (2, 3). Observations on veal calves in France revealed a mortality rate of almost 8% originating from umbilical infections (4).

The umbilical structures of a bovine fetus comprise two umbilical veins, uniting in range of the inner umbilical ring, two umbilical arteries and the urachus (5). The umbilical cord ruptures during or shortly after expulsion of the calf (6).

Over the course of the first 5 days (7), the umbilical cord mummifies and subsequently detaches in the first 2 weeks of life (8), but still provides a port of entry for potential pathogens. Umbilical infections are most commonly caused by invasion of opportunistic, in particular fecal, cutaneous, mucosal and environmental bacteria, and are primarily observed in the first 3 weeks of a calf’s life (3, 4, 9).

Septicemia characterized by the systemic inflammatory response syndrome as well as invasion and damage of various organs (e.g., liver, lung, joints, brain) can develop as a result of omphalitis (6).

Besides exhibiting a higher risk of carcass condemnation in veal production (10), calves with umbilical infections show multiple signs of impaired well-being. Shorter lying times indicating abdominal pain (11) and reduced daily body weight gain (12) as well as an increased risk of suffering from additional diseases are common (13).

Calving management, initial care of the neonate (e.g., colostrum management, navel disinfection) and housing conditions of the calf during the first weeks of life have been shown to play a central role in the occurrence of omphalitis in dairy calves (14–16).

Evaluating the role of these risk factors is an important step in supporting farmers from an animal welfare perspective as well as an economical perspective. Evidence-based guidelines for farmers, veterinarians and consultants can be developed as a result. The aim of this study was to identify risk factors for omphalitis, particularly in the domains of calving management and dam health as well as initial care, housing conditions and nutritional management in the context of a cross-sectional field study including a greater number of farms and calves.

## 2 Materials and methods

All the information analyzed in the present study was retrieved from five sources: PraeRi (17) interviews, evaluations of the calves’ housing, clinical examinations of calves, examinations of dams and institutional databases. The potential risk factors analyzed for omphalitis in neonatal dairy calves are provided in Table 1.

**Table 1:** Variables selected for confounder adjusted statistical analyses as potential risk factors for omphalitis in neonatal dairy calves from German dairy farms.

Calving management^1^	Navel disinfection^1^	Calf feeding^1^	Calf housing in the first 2 weeks of life^2^	Calf related factors	Dam related factors
Calving area: usual husbandry	Routine navel disinfection	Amount of first colostrum offered at first feeding	Housing type	Abdominal hygiene score^2^	Body condition score^2^
Calving area: single pen		Amount of milk offered daily to calves aged 0–14 days	Nesting score	Multiples^3^	Average age of primiparous cows at first calving^3^
Calving area: group pen			Cleanliness of the lying area		Gestation time^3^
Calving area: combined pen for calving and diseased cows			Dampness of the lying area		
Calving area: pasture					
General time calves spent with dam after birth					

1 Data retrieved from questionnaire.

2 Data retrieved from clinical examination or evaluation of housing conditions of calves.

3 Data retrieved from databases.

### 2.1 PraeRi dataset and interview with the farmer/herd manager

The data set used originates from the results of a cross-sectional study in Germany [“PraeRi” (17)] performed to evaluate the present situation on dairy farms regarding animal health, husbandry conditions, hygiene, nutrition, and management practices. Between December 2016 and July 2019, a team of specifically trained veterinarians performed in-depth farm visits on 765 dairy farms in seven federal states belonging to three structurally different regions in Germany: North (N) (Schleswig-Holstein and Lower Saxony), East (E) (Mecklenburg-Western Pomerania, Brandenburg, Saxony-Anhalt, Thuringia) and South (S) (Bavaria).

Each dairy farm was visited once, and data was collected according to a standardized protocol. Each farm visit included an interview with the farmer or herd manager, clinical examinations of a pre-defined sample size of animals and an evaluation of housing facilities. Once a year, trainings were performed to evaluate inter-observer reliability of observations made by the study veterinarians and to fine-tune agreements between the results of the clinical examination of different observers. Sample size calculations of farms, recruitment methods and the implementation of the inter-observer comparisons are described in detail by Merle et al. (18). The entire questionnaire is available in German on www.praeri.de and can be requested in German or English from the authors.

In the present study, answers to 48 questions relevant to the complex of umbilical health in calves were selected from the PraeRi dataset addressing the following aspects: farm management, calving management, husbandry conditions of calves, colostrum management, feeding management until weaning as well as questions of prophylactic measures, and health of calves and cows in the transition period.

One multiple-choice question addressed the main calving area in the last 12 months preceding the farm visit (possible answers: usual husbandry, single pen, group pen, combined pen for calving and diseased cows, pasture). For further analyses the multiple-choice answers were transformed into five binominal choice questions (e.g., calving area: usual husbandry—yes or no).

### 2.2 Evaluation of housing conditions of the calves

In Germany, most farms keep calves in single boxes in the first 2 or 3 weeks of life, while older calves are kept in group boxes until weaning. Therefore, in the PraeRi study, calves were categorized by age and separate assessment sheets were filled out for those aged less than 2 weeks of age and those aged over 2 weeks. As most umbilical infections occur in the first 3 weeks of life (3) and mostly develop at or shortly after birth, evaluations for older calves were neglectable for this analysis. Hence, only evaluations of housing conditions of calves in their first 2 weeks of life were included.

Housing conditions and hygiene of each compartment in every stable of the calves were evaluated. Each housing facility (single and group boxes) was assessed for dampness of the bedding and contamination with urine and feces. At the farm visit an average dampness and average contamination score of all lying surfaces was assigned to each age group. The average nesting score was calculated from observing a sample of three calves per group (19).

### 2.3 Selection of calves and clinical examination

The number of calves per farm, that were clinically examined, was calculated based on the number of pre-weaned calves (aged maximum 6 months) present on the farm on the day of the farm visit. The sample calculation is described in detail by Merle et al. (18). The region-specific cut-off values are shown in Table 2.

**Table 2:** Region-specific cut-off values for the number of calves examined on each German dairy farm based on the number of pre-weaned calves housed on the farm on the day of the farm visit.

Region	Number of pre-weaned calves on the farm	Number of pre-weaned calves examined
North	1–54 ≥ 55	All pre-weaned calves 54 (random sample)
East	1–40 41–73 ≥ 74	All pre-weaned calves 40 (random sample) 73 (random sample)
South	1–33 ≥ 34	All pre-weaned calves 33 (random sample)

Sample calculation based on the expected prevalence of 40%^*^, confidence level: 95%, power: 80%, precision: ±5%. ^*^ Sample calculation for the whole PraeRi study. Therefore, the highest prevalence of all diseases addressed in the study was estimated from literature (prevalence of lameness).

If the required sample size of calves was smaller than the number of calves present on the farm on the day of the visit, the calves selected were equally distributed between the different age and husbandry groups. Therefore, for each group, the same percentage of calves was randomly selected for clinical examination (e.g., 80% of all calves in single housing, 80% of all calves in group 1, 80% of all calves in group 2, …). Within the different groups the calves undergoing a clinical examination were chosen by chance. Both, female and male calves were included. Calves that had been examined were marked to avoid repeated examinations. For identification of the animals, the last five digits of the ear tag were recorded. Findings were noted on a separate sheet.

Inspection and palpation of the external navel was performed to determine the health status of the umbilicus. Presence of inflammatory symptoms at the umbilicus such as swelling with or without a pain response on palpation, reddening or warmth was diagnosed as omphalitis. Umbilical hernias were noted separately but were not considered for further investigations. Haircoat and skin of the calves were evaluated for fecal soiling according to the hygiene score described by Kellermann et al. (20). For this purpose, the hair coat and skin at the from lateral visible abdominal wall extending from the elbow to the knee fold were assessed. Only older, dried, permanent dirt was considered.

### 2.4 Selection and examinations of dams

On each farm visit, a defined number of cows was evaluated by the veterinarians. As not every cow could be examined, only a certain number of cows was scored by chance. The sample calculation is specified by Merle et al. (18).

To evaluate risk factors for omphalitis in calves, selected cow-related parameters were analyzed.

Each cow was assigned a body condition score (BCS) using the scoring system from Edmonson et al. (21) modified by Metzner et al. (22). On basis of their BCS cows were assigned to one of the following categories: under- (< 2.75 for Holstein + Brown Swiss and < 3.25 for Simmental), optimal and over-conditioned (> 3.75 for Holstein + Brown Swiss and > 4.25 for Simmental cows) (23–25).

To show a possible association between the chosen parameters and the occurrence of omphalitis, only cows, whose calves had been included were comprised in the final analysis.

### 2.5 Institutional databases

Further information was retrieved from databases as the National Traceability and Information System for Animals (“Herkunftssicherungs- und Informationssystem für Tiere,” HIT) and the national milk recording system (DHI), respectively. Information retrieved from HIT was: age and sex of calves, farm size (as number of lactating and dry cows), dam’s breed and age of first calving from primiparous cows. From DHI the main information retrieved was: parity, milk yield, gestation length and the birth of multiples.

Median milk yield in the past 12 months preceding the farm visit was expressed as Energy Corrected Milk yield (ECM). ECM was calculated as described by Spiekers et al. (26).

### 2.6 Data analysis

The full PraeRi data set was filtered for relevant information regarding omphalitis in neonatal dairy calves. The original data set contained data of calves from birth until weaning (aged maximum 6 months). Prior to data analyses, we looked at the age distribution of calves diagnosed with omphalitis. In the first 4 days of life a peak of calves showing signs of omphalitis was present (predominantly swelling of the umbilicus). The prevalence of omphalitis significantly declined after the 5th day of life. In this study one sign of inflammation, such as a swollen umbilicus on its own, was already diagnosed as omphalitis. In the first days of life, however, a swollen navel might be physiological or might have other causes than omphalitis (e.g., hematoma). Therefore, a correct diagnosis of omphalitis is difficult. Hence, we excluded calves younger than 5 days to minimize the number of false positive cases. As most umbilical infections occur in the first 3 weeks of life (3) and the focus of this analysis were risk factors for developing an omphalitis, the analyses focused on calves aged from five to 21 days. A description of the study population is shown in Table 3.

**Table 3:** Omphalitis prevalence and description of the study population from 567 German dairy farms with regional differences for all three regions.

Variable	North	East	South	Overall
Omphalitis				
Omphalitis (animal level) % (n)				
Yes	50.5 (368)	27.8 (613)	16.2 (83)	30.9 (1,064)
No	49.5 (361)	72.2 (1,590)	83.8 (430)	69.1 (2,381)
Omphalitis (farm level) % (n)				
Median IQR	50.0 (180) 21.1–70.7	25.5 (216) 13.4–37.5	0.0 (171) 0.0–25.0	25.0 (567) 0.0–50.0
Study population (animal level)				
Sex % (n)				
Male	36.8 (268)	36.6 (806)	46.2 (237)	38.1 (1,311)
Female	63.2 (461)	63.4 (1,397)	53.8 (276)	61.9 (2,134)
Breed % (n)				
Holstein^1^	85.8 (615)	89.4 (1,967)	4.7 (24)	76.0 (2,606)
Simmental	2.0 (14)	0.3 (6)	84.0 (431)	13.1 (451)
Cross breed between dairy and beef/dual purpose breed with emphasis on beef^2^	9.6 (69)	7.0 (154)	6.0 (31)	7.4 (254)
Other dairy breeds and their cross breeds^3^	2.6 (19)	3.3 (73)	5.3 (27)	3.5 (119)
Study population (farm level)				
Farm size (number of lactating and dry cows)				
Median (number of farms)	101 (180)	180 (216)	46 (171)	104 (567)
IQR	65–137	150–299	8–66	55–170
Farming type % (n)				
Conventional	93.9 (169)	93.5 (202)	84.2 (144)	90.8 (515)
Organic or in transition	6.1 (11)	6.5 (14)	15.8 (27)	9.2 (52)
Energy corrected milk yield				
Median (number of farms)	28.9 kg (174)	29.4 kg (216)	25.1 kg (160)	28.1 kg (550)
IQR	26.0–31.0 kg	26.9–31.6 kg	22.6–27.5 kg	25.0–30.5 kg

1 Black and Red Holstein.

2 Cross breed between dairy and beef breed or 2 beef breeds, Pinzgauer cattle, other.

3 Brown Swiss, Jersey, German black pied cattle, German red pied cattle, Angler, cross breed between 2 dairy breeds.

IQR, Interquartile Range.

For the statistical analyses, a list of all potential risk factors for omphalitis in calves that were assessed during the farm visits was made based on literature research, expert knowledge and expected influence on omphalitis (e.g., from the interview, housing, or animal evaluation).

For each variable, descriptive analyses were performed on farm or animal level with SAS 9.4 (SAS Institute, Cary, North Carolina, United States). If a variable did not show normal distribution, it was categorized, or the logarithm of the variable was used. If one category in a categorical outcome included less than 5% observations, the categories were revised or, if not possible, the variable was excluded from further analyses.

For the remaining variables univariable logistic regression models were performed concerning the association of each variable with omphalitis.

For the multivariable analyses a hypotheses-based approach was used. Prior to the analyses, hypotheses were formulated with the target variable “omphalitis” and all possible influence variables that were evaluated in the PraeRi study (e.g., “If a navel disinfection is used after birth, the omphalitis prevalence is lower.”). Based on those formulated hypotheses and if the potential risk factor could be addressed by farmers, 18 variables were selected for further investigation. Most of these 18 variables chosen were suspected risk factors for omphalitis reported previously in the literature (e.g., calving pen, colostrum management, housing conditions of the calves). The other factors were chosen based on the results of the univariable analyses (*p* < 0.1) (e.g., BCS of the dam) or because of a presumed influence on omphalitis (e.g., gestation time). A list of all 18 variables is shown in Table 1.

To identify the possible confounder variables for omphalitis all variables from our formulated hypotheses were put into one large overview causal diagram. We used a causal directed acyclic graph (DAG)^1^ with “omphalitis” as target variable. Arrows between different variables were drawn to demonstrate the connections between all variables included. In the next step we build individual DAGs for each potential risk factor as influence and with “omphalitis” as target variable. The individual DAGs were reduced by variables that showed no connection between target and influence variable, marked automatically by the program “dagitty.” Farm size, farming type, and region were regarded as general confounder variables and therefore included in each model. Further possible confounder variables were marked by the program based on the arrows drawn. All causal diagrams used are presented as Supplementary Figures S1–S18. The individual confounder variables for each model are listed in the footnotes of the respective model (Tables 4– [Table tab5] [Table tab6] [Table tab7] [Table tab8] 9).

**Table 4:** Description, univariable and confounder adjusted analyses of potential risk factors for omphalitis in calves from 567 German dairy farms concerning calving management for all three regions on farm level.

Univariable analysis							Confounder adjusted model				
Variable	Number of farms (%)	Crude estimate	OR	95% CI		*p*-value	Adjusted estimate	IRR	95% CI		*p*-value
Calving area: usual husbandry^1^^,^^2^						**0.005**					**0.636**
No	477 (84.4)	Reference	.	.	.	.	Reference	.	.	.	.
Yes	88 (15.6)	−0.387	0.68	0.51	0.90	0.008	−0.072	0.93	0.69	1.25	0.638
Calving area: single pen^1^						**0.884**					**0.219**
No	450 (79.6)	Reference	.	.	.	.	Reference	.	.	.	.
Yes	115 (20.4)	−0.013	0.99	0.83	1.18	0.884	−0.1	0.90	0.77	1.06	0.223
Calving area: group pen^1^						**0.948**					**0.576**
No	268 (47.4)	Reference	.	.	.	.	Reference	.	.	.	.
Yes	297 (52.6)	0.005	1.01	0.87	1.17	0.948	0.043	1.04	0.90	1.21	0.576
Calving area: combined pen for calving and diseased cows^1^						**0.034**					**0.342**
No	486 (85.9)	Reference	.	.	.	.	Reference	.	.	.	.
Yes	80 (14.1)	0.236	1.27	1.02	1.57	0.03	0.104	1.11	0.90	1.37	0.338
Calving area: pasture^1^						**0.088**					**0.514**
No	526 (92.9)	Reference	.	.	.	.	Reference	.	.	.	.
Yes	40 (7.1)	0.264	1.30	0.97	1.75	0.08	0.1	1.11	0.82	1.49	0.51
General time calves spent with dam after birth^1^^,^^3^						**0.095**					**0.185**
Removed immediately	306 (54.7)	Reference	.	.	.	.	Reference	.	.	.	.
Up to 8 h	128 (22.9)	0.047	1.05	0.85	1.29	0.66	−0.03	0.97	0.80	1.18	0.758
8 to 12 h	59 (10.6)	0.297	1.35	1.05	1.73	0.021	0.009	1.01	0.79	1.28	0.944
More than 12 h	66 (11.8)	−0.092	0.91	0.7	1.19	0.504	−0.281	0.75	0.58	0.99	0.04

1 Adjusted for farm size, region, farming type.

2 Adjusted for highest academic degree of the interviewee, reported incidence of milk fever.

3 Adjusted for regular participation of the interviewee in advanced training courses, calving area (usual husbandry, single pen, group pen, combined pen for calving and diseased cows, pasture).

OR, odds ratio; IRR, incidence risk ratio; 95% CI, 95% confidence interval.Bold values: global *p*-value of the respective model.

**Table 5 tab5:** Description, univariable and confounder adjusted analyses of potential risk factors for omphalitis in calves from 567 German dairy farms concerning navel disinfection for all three regions on farm level.

Univariable analysis							Confounder adjusted model				
Variable	Number of farms (%)	Crude estimate	OR	95% CI		*p*-value	Adjusted estimate	IRR	95% CI		*p*-value
Navel disinfection^1^						**0.033**					**0.202**
Never	262 (46.5)	Reference	.	.	.	.	Reference	.	.	.	.
Always (> 90% of calvings)	219 (38.8)	−0.218	0.8	0.68	0.94	0.009	−0.144	0.87	0.72	1.03	0.112
Sometimes	83 (14.7)	−0.105	0.9	0.71	1.14	0.393	−0.157	0.85	0.68	1.08	0.185

1 Adjusted for farm size, region, farming type.

OR, odds ratio; IRR, incidence rate ratio; 95% CI, 95% confidence interval.Bold values: global *p*-value of the respective model.

**Table 6 tab6:** Description, univariable and confounder adjusted analyses of potential risk factors for omphalitis in calves from 567 German dairy farms concerning calf feeding for all three regions on farm level.

Univariable analysis							Confounder adjusted model				
Variable	Number of farms (%)	Crude estimate	OR	95% CI		*p*-value	Adjusted estimate	IRR	95% CI		*p*-value
Amount of colostrum offered at first feeding^1^^,^^2^						**0.832**					**0.653**
Up to 2.5 liters	149 (33.1)	Reference	.	.	.	.	Reference	.	.	.	.
2.5 to 3.5 liters	192 (42.7)	0.057	1.06	0.87	1.28	0.562	0.0154	1.02	0.85	1.21	0.864
More than 3.5 liters	109 (24.2)	0.050	1.05	0.85	1.31	0.652	−0.068	0.93	0.77	1.14	0.5
Amount of milk offered daily to each calf aged 0–14 days^1^^,^^3^						**0.51**					**0.470**
*Ad libitum*	85 (15.1)	Reference	.	.	.	.	Reference	.	.	.	.
Up to 6 liters	256 (45.5)	−0.135	0.87	0.70	1.09	0.225	−0.157	0.86	0.71	1.04	0.112
6 to 8 liters	139 (24.7)	−0.151	0.86	0.68	1.09	0.211	−0.121	0.89	0.72	1.09	0.253
More than 8 liters	83 (14.7)	−0.034	0.97	0.74	1.27	0.808	−0.095	0.91	0.71	1.16	0.445

1 Adjusted for farm size, region, farming type.

2 Adjusted for calving area (usual husbandry, single pen, group pen, combined pen for calving and diseased cows, pasture), general time calves spent with the dam after birth, standard operation protocol for prophylactic measures for calves.

3 Adjusted for general time calves spent with the dam after calving, same care for male and female calves.

OR, odds ratio; IRR, incidence rate ratio; 95% CI, 95% confidence interval.Bold values: global *p*-value of the respective model.

**Table 7 tab7:** Description, univariable and confounder adjusted analyses of potential risk factors for omphalitis in calves from 567 German dairy farms concerning calf housing for all three regions on farm level.

Univariable analysis							Confounder adjusted model				
Variable	Number of farms (%)	Crude estimate	OR	95% CI		*p*-value	Adjusted estimate	IRR	95% CI		*p*-value
Housing type in the first 2 weeks of life^1^						**0.722**					**0.729**
Group box or hutch	46 (8.4)	Reference	.	.	.	.	Reference	.	.	.	.
Single box or hutch	497 (90.7)	−0.112	0.89	0.68	1.18	0.426	−0.088	0.92	0.71	1.18	0.502
Other	5 (0.9)	−0.168	0.85	0.38	1.87	0.679	−0.222	0.80	0.40	1.589	0.525
Nesting score in the first 2 weeks of life^1^^,^^2^^,^^3^						**0.603**					**0.965**
Sparsely interspersed	143 (27.9)	Reference	.	.	.	.	Reference	.	.	.	.
Moderatly interspersed	277 (54.0)	−0.064	0.94	0.78	1.13	0.504	−0.022	0.98	0.82	1.16	0.804
Well interspersed	93 (18.1)	−0.121	0.89	0.70	1.13	0.32	−0.025	0.98	0.78	1.22	0.827
Clean lying area in the first 2 weeks of life^1^^,^^4^^,^^5^						**0.103**					**0.259**
No	59 (10.8)	Reference	.	.	.	.	Reference	.	.	.	.
Yes	374 (68.6)	−0.205	0.81	0.64	1.04	0.097	−0.14	0.87	0.68	1.11	0.262
Dry lying area in the first 2 weeks of life^1^^,^^6^^,^^7^						**0.249**					**0.001**
No	171 (31.4)	Reference	.	.	.	.	Reference	.	.	.	.
Yes	374 (68.6)	−0.113	0.89	0.77	1.04	0.245	−0.257	0.77	0.66	0.9	0.001

1 Adjusted for farm size, region, farming type.

2 Adjusted for housing type in the first 2 weeks of life, same care for male and female calves.

3 “Sparsely interspersed” (limbs completely visible, calf lies on top of the bedding); “moderately interspersed” (parts of the limbs visible, calf is partly embedded in the bedding); “well interspersed” (no visible limbs, calf is well embedded in the bedding).

4 Adjusted for housing type in the first 2 weeks of life, nesting score in the first 2 weeks of life, dry lying areas in the first 2 weeks of life, amount of milk offered daily to each calf aged 0–14 days, same care for male and female calves, standard operation protocol for prophylactic measures for calves, treatment incidence for diarrhea in calves.

5 Clean = “yes” (clean or with single piles of feces); clean = “no” (some percentages of the area are dirty or completely covered in feces).

6 Adjusted for housing type in the first 2 weeks of life, nesting score in the first 2 weeks of life, amount of milk offered daily to each calf aged 0–14 days, access to water in the first 2 weeks of life, same care for male and female calves, treatment incidence for diarrhea in calves.

7 Dry = “yes” (dry); dry = “no” (moist in some places or > 50% of the area is moist or wet).

OR, odds ratio; IRR, incidence rate ratio; 95% CI, 95% confidence interval.Bold values: global *p*-value of the respective model.

**Table 8 tab8:** Description, univariable and confounder adjusted analyses of potential risk factors for omphalitis in calves from 567 German dairy farms concerning the calf for all three regions on animal level.

Univariable analysis							Confounder adjusted model				
Variable	Number of calves (%)	Crude estimate	OR	95% CI		*p*-value	Adjusted estimate	OR	95% CI		*p*-value
Abdominal hygiene score^1^^,^^2^^,^^3^											**0.424**
Absent or slight soiled	2,880 (96.1)	Did not converge					Reference	.	.	.	.
Moderate or severe soiling	118 (3.9)						0.173	1.19	0.78	1.81	0.424
Multiples^1^^,^^4^						**0.895**					**0.958**
Yes	173 (5.3)	Reference	.	.	.	.	Reference	.	.	.	.
No	3,082 (94.7)	0.031	1.03	0.65	1.64	0.895	−0.013	0.99	0.62	1.58	0.958
Sex						**< 0.0001**					
Female	2,134 (61.9)	Reference	.	.	.	.	Not further investigated				
Male	1,311 (38.1)	0.331	1.39	1.20	1.61	< 0.0001					

1 Adjusted for farm size, region, farming type.

2 Adjusted for calving area (usual husbandry, single pen, group pen, combined pen for calving and diseased cows, pasture), general time calves spent with the dam after birth, housing type in the first 2 weeks of life, nesting score in the first 2 weeks of life, dry lying areas in the first 2 weeks of life, clean lying areas in the first 2 weeks of life.

3 “Absent or slight soiling” (soiling of ≤10% of the abdominal surface); “moderate or severe soiling” (soiling of 10–30% of the abdominal surface + soiling of >30% of the abdominal surface).

4 Adjusted for highest academic degree of the interviewee, median Energy Corrected Milk of the farm, breeding goal: calving ease, parity of the cow, body condition score of the dam, breed of the calf.

OR, odds ratio; IRR, incidence risk ratio; 95% CI, 95% confidence interval.Bold values: global *p*-value of the respective model.

**Table 9:** Description, univariable and confounder adjusted analyses of potential risk factors for omphalitis in calves from 567 German dairy farms concerning the dam for all three regions on animal level.

Univariable analysis							Confounder adjusted model				
Variable	Number of dams (%)	Crude estimate	OR	95% CI		*p*-value	Adjusted estimate	OR	95% CI		*p*-value
Body condition score^1^^,^^2^						**0.0008**					**0.026**
Optimal conditioned	970 (43.9)	Reference	.	.	.	.	Reference	.	.	.	.
Under-conditioned	714 (32.3)	0.488	1.63	1.26	2.1	0.002	0.321	1.38	1.06	1.79	0.016
Over-conditioned	527 (23.8)	0.25	1.29	0.95	1.73	0.101	0.313	1.37	1.00	1.86	0.045
Age at first calving of primipara^1^^,^^3^						**0.431**					**0.645**
Age in days (median, n) (IQR)	788 (1,191) (741–858)	0.0006	1.00	1.00	1.00	.	0.0004	1.00	1.00	1.00	.
Gestation time^1^^,^^4^						**0.277**					**0.543**
Gestation time in days (median, n) (IQR)	278 (2,325) (275–282)	0.008	1.01	0.99	1.02	.	0.005	1.01	0.99	1.02	.

1 Adjusted for farm size, region, farming type.

2 Adjusted for highest academic degree of the interviewee, regular participation of the interviewee in advanced training courses, type of enterprise, dry cow feeding, feeding of anionic salts, use of vitamin D3 injections.

3 Adjusted for highest academic degree of the interviewee, breeding goal: calving ease, breed of the calf.

4 Adjusted for breed of the calf, primipara/multipara, farm ID.

OR, odds ratio; IRR, incidence risk ratio; 95% CI, 95% confidence interval.Bold values: global *p*-value of the respective model.

Based on these DAGs, confounder adjusted statistical analyses with risk for omphalitis as dependent variable on animal level were performed with SAS. One model for each respective risk factor with all possible confounder variables as independent variables was built. Some influence variables were analyzed on farm level (e.g., data from interviews), others on animal level (e.g., data from dam and calf examinations). If the potential risk factor was on animal level, a mixed logistic regression model was used with farm as random factor. The model including the gestation time of the dam as influence factor, however, did not converge. Therefore, a mixed logistic regression model with farm as fixed factor was used. If the influence factor was on farm level, a generalized linear regression model with negative binomial distribution and logit link of farm level data was adapted with the number of calves with omphalitis as dependent variable and the number of calves investigated as offset variable.

Odds ratios (OR, logistic regression) or incidence rate ratios (IRR, generalized linear regression) including 95% confidence intervals (95% CI) were calculated for the respective influence factor. Differences were considered significant at *p* < 0.05.

## 3 Results

### 3.1 Study population

The description of the study population is presented in Table 3. A total of 3,445 calves from 567 farms were included in the final analysis. The size of the farms varied between regions with the largest farms located in the East of the country and the smallest farms in the South. The most common breed overall was Holstein (76.0%). Simmental was the predominant breed in the South (84.0%).

Most of the farms were run conventionally (90.8%). The remaining farms (9.2%) were either organic or in transition to become organic. The highest proportion of organic farms was situated in the South.

### 3.2 Prevalence of omphalitis

Overall, 1,064 out of the 3,445 calves were diagnosed with omphalitis based on the pre-set criteria (30.9%; 95% CI: 29.3–32.5%). The median farm-level prevalence across all three regions was similar to the prevalence on animal level with significant regional differences (*p* ≤ 0.0001, median: 25.0%; IQR: 0.0–50.0%; Supplementary Table S1). The highest median prevalence on farm level was observed in the North with 50.0% (E: 25.5%, S: 0.0%).

Over all three regions farmers reported to have treated 4.5% of their calves for omphalitis within the past 12 months preceding the farm visit (median).

Out of 18 potential risk factors considered in the confounder adjusted analyses, three factors showed an influence on the risk of dairy calves developing omphalitis: the general time calves spent with the dam after birth, dry lying areas in the first 2 weeks of life and the body condition score of the dam. All results of the univariable and confounder adjusted analyses of potential risk factors for omphalitis are shown in Tables 4–9. The results of the univariable analyses of the main confounder variables are shown in Supplementary Tables S1, S2.

### 3.3 Farm-level risk factors for omphalitis

The description over all three regions of the analyzed potential risk factors on farm level are depicted in Tables 4–7.

#### 3.3.1 Calving management

In the South, the majority of farmers reported that cows were not separated for calving from their usual husbandry (38.0%). In North and East, 5.6 and 6.0% of farmers reported that no separate calving pen was provided, respectively. Pasture calvings were more common in the North (11.7%), than in the South (5.3%) and East (4.6%). While some of the univariable analyses related to the calving management (calving area, general time calves spent with the dam after birth) showed an association between potential influence factors and the prevalence of omphalitis, none of the confounder adjusted models resulted in a statistically significant association, but tendencies could be shown. Calves from farms that separated calf and dam after more than 12 h showed a lower risk of omphalitis than calves from farms that separated calf and dam immediately after birth (IRR = 0.75, 95% CI: 0.58–0.99) (Table 4).

#### 3.3.2 Navel disinfection

More than one third of the farmers stated that navel disinfection was performed in more than 90% of all calves (38.8%) born. While 14.7% of farmers reported sporadic navel disinfection, 46.5% of farms did not carry out navel disinfection at all. The multivariable analysis including navel disinfection as potential risk factor of omphalitis, showed no statistical association between both variables (Table 5).

#### 3.3.3 Calf feeding

No effect of calves’ feeding was detected on the prevalence of omphalitis in our data set (Table 6).

#### 3.3.4 Calf housing

Most farms provided clean and dry lying areas for the calves in the first 2 weeks of life (clean: 89.2% and dry: 68.6%, respectively). The highest number of farms with contaminated or moist lying areas was observed in the East. Calves from farms providing predominantly dry lying areas had a lower risk of developing omphalitis than calves from farms with predominantly wet lying areas (IRR = 0.77, *p* = 0.001, 95% CI: 0.66–0.90) (Table 7).

#### 3.3.5 Farming type

On organic farms or farms transitioning to an organic system, the risk of calves developing omphalitis was 0.69 times smaller than on conventional farms (*p* = 0.035, Supplementary Table S1).

### 3.4 Animal-level risk factors for omphalitis

The description over all three regions of the analyzed potential risk factors on animal level are described in Tables 8, 9.

Results from the univariable analyses showed that the risk of male calves suffering from omphalitis was 1.39 times higher than the risk of female calves (Table 8).

The body condition score of 2,211 dams was analyzed. Less than half of all dams across all regions had a BCS within the target range between day five and 21 postpartum (43.9%). Overall, 23.8% of dams were over-conditioned with the highest percentage found in the East (33.0%). On farms in the North, half of the dams were under-conditioned (50.4%). Calves born to over- or under-conditioned dams were 1.4 times more likely to develop omphalitis, respectively, compared to calves from optimal conditioned dams (OR = 1.37/1.38, *p* = 0.026) (Table 9).

## 4 Discussion

After confounder-adjusted statistical analyses of 18 selected variables, the dampness of the lying area in the first 2 weeks of life, the body condition score of the dam five to 21 days in milk (DIM) and the general time calves spent with the dam after birth were confirmed as risk factors for omphalitis in neonatal dairy calves.

The high prevalence of symptoms of omphalitis in dairy calves observed in the present study highlights the importance of research in this field of calf health for reasons of animal welfare. The prevalence of omphalitis observed is in accordance with other recently published studies (3, 11, 27).

A strength of this study is the high number of calves examined (*n* = 3,445) and the variety in types of farms located in different regions of Germany as well as the opportunity to consider selected characteristics of related dams. The prevalence of omphalitis differed significantly between regions, which may be explained by the different farming systems and management styles common in the respective regions. The study population includes the main age at risk for omphalitis in calves, as most umbilical infections occur in the first 3 weeks of life (3), even though some umbilical infections might be missed due to exclusion of calves younger than 5 days of age.

In this study, clinical findings were used to assign calves to one of two categories: “omphalitis” or “healthy.” Due to the stringent assignment to either group on basis of clinical findings, we cannot conclude that each calf in the omphalitis category required antibiotic treatment.

However, the discrepancy between the number of calves showing signs of omphalitis per farm and the small number of omphalitis treatments reported by farmers is remarkable. While every fourth calf aged five to 21 days presented with signs of omphalitis, the median reported treatment incidence for omphalitis was 4.5% in the 12 months preceding the farm visit. It is important to note that calf health parameters are often not fully documented. Hence, under- or overestimation regarding the numbers of calves treated for omphalitis cannot be ruled out. Nonetheless, these findings highlight the importance of including umbilical health in veterinary advisory activities, as well as improving detailed documentation concerning calves’ health. Nevertheless, the conclusion should not be to treat a quarter of calves on the farm, but to reduce the omphalitis prevalence overall.

To achieve this goal, farm personnel must be trained to detect umbilical inflammation correctly and early on. In addition, steps must be taken to promote awareness of risk factors on farms as well as providing guidance on how to improve umbilical health.

### 4.1 Farm-level factors

#### 4.1.1 Calving management

Although some of the univariable analysis of the calving area were significant, after adding all confounders, no association between calving area and the occurrence of omphalitis was confirmed. Even though the calving area was not proven to increase the risk for omphalitis in this study, regardless the kind of calving space itself, the management of the calving area may have a greater influence on the calf’s health, particularly its hygienic conditions. For instance, the hygienic conditions in a well-managed and regularly cleaned group box are likely to be superior to those in a single calving pen which is cleaned only once a month (28). A recent study in France regarding beef calves determined the wetness of the bedding in the calving area as risk factor for omphalitis (27). In this study, however, the kind of the intended calving area was asked by questionnaire. As not every calving takes place in the intended calving area (29), future studies should include more detailed information, especially considering the hygienic conditions of the actual calving area.

After adding all confounders, the confounder adjusted analysis showed that on farms where calves stayed with their dam for more than 12 h, calves tended to have a reduced risk for omphalitis compared to farms with immediate separation. The timing of cow-calf separation is a consistently heavily discussed topic. Various studies show that calves that stay with the dam for a longer time are less prone to diseases (30, 31) and have greater weight gains than calves that are separated immediately after birth (32). In addition, calves that stay longer with their dams can suckle regularly, which is thought to improve the absorption of immunoglobulins from the ingested colostrum (33). Furthermore, calves are more motivated to repeatedly stand up to suckle, which may promote umbilical cord drying. Other studies, however, describe negative effects on calves’ health. A delayed separation is associated with increased emotional trauma (31), which is relevant from an animal welfare perspective. In addition, longer exposure of the neonate to pathogens from the dam can increase the risk for morbidity and mortality (34). Nursed calves tend to have lower blood immunoglobulin concentrations than hand-fed calves (30), often due to a calf’s inability to rise and suck from the dam within the first 6 h after birth (35) and/or the absence of monitoring measures to ensure that each calf receives the correct amount of good quality colostrum (36). Hence, if calves that stay with their dams are not monitored closely to ensure colostrum intake within the first 6 h after birth, failure of passive transfer may occur. Other studies have revealed no effect of time of separation on calf health (37). Therefore, the recommendation for or against immediate separation must take the conditions on each individual farm into consideration. Delaying separation in order to achieve the benefits described above should only be recommended on farms where hygienic calving conditions can be ensured (33).

#### 4.1.2 Navel disinfection

Results from the confounder adjusted analysis showed no association between the routine application of navel disinfection and the prevalence of omphalitis in neonatal dairy calves. In less than 40% of the farms included in this study, navel disinfection was performed on all new-born calves as routine measure. The efficiency of navel disinfection is an ongoing topic of intense discussion. Reports from different countries on application of routine navel disinfection in new-born calves vary between 34.9% (Uruguay) and 88.2% (Czech Republic) (38, 39). In a German study two-thirds out of 42 farms performed navel disinfection (40).

The prophylactic effect of navel disinfection on umbilical health (41–43), calf health in general and animal welfare (33, 44) is commonly stated. Nonetheless, other studies have found no positive influence of navel disinfection on umbilical health (45–47) or even a negative association between disinfection and the occurrence of respiratory diseases (48). These results must be interpreted cautiously. Most studies did not include a control group while comparing different disinfectants and ways of disinfection. Furthermore, some observation periods were very short [e.g., a single follow-up examination 24 h post-natum (46)], so no conclusions about the long-term effects can be drawn. Given these contrary findings and the fact that forced manipulation of the umbilical cord has been associated with additional umbilical health problems (49), farmers will have to consider carefully whether navel disinfection is indicated based on the thorough control of the umbilical health status of their calves. Farms with less than 5% of calves with umbilical infections or hernias should focus on hygiene of the calving area and correct colostrum management. On farms with pronounced umbilical health issues in calves (> 5% umbilical infections and hernias), repeated routine disinfection of the navel is recommended additionally (6, 33). In this study, all information regarding handling of new-born calves was derived from interviews with the farmers or herd managers and the exact method of navel disinfection (e.g., disinfectant used, method of application, duration between birth and disinfection) was not enquired. As no on-farm observations of the navel disinfection were conducted, the exact steps could not be verified by the authors. However, in accordance with observations from other studies, the results indicate that navel disinfection cannot make up for flaws in management of the neonate.

#### 4.1.3 Calf feeding

The amount of first colostrum had no significant impact on the occurrence of omphalitis. Our findings are in accordance with a recent study in beef calves that did not find a statistical association between the omphalitis prevalence and failure of passive immunity transfer (50). Regardless these results, the colostrum management is the main factor for a healthy calf (51). Other studies, however, described the influence of an adequate colostrum feeding on the risk of umbilical infections (43, 51). The design of this study was not suitable to evaluate the influence of the colostrum management on umbilical health. The information about the management decision of the quantity of first colostrum was requested by questionnaire, but neither the quality of the colostrum fed or the time of first feeding, nor exact data on the individual supply to individual calves were included. These aspects play a major role in the serum immunoglobulin concentration in calves (51), while in this study the amount of colostrum *per se* did not influence the risk for omphalitis.

#### 4.1.4 Calf housing

This study revealed that calves from farms with predominantly dry lying areas had a lower risk for omphalitis compared to calves from farms with predominantly wet lying areas. A recent study including beef calves, however, could not confirm the wetness of the calves’ pen bedding as a risk factor for omphalitis (27).

Nevertheless, feces and urine function as carriers for pathogens (52) and neonatal calves spend more than 70% of the time recumbent (53). Therefore, soiled bedding poses a risk for animal health and welfare (44). Due to legal regulations in Germany (54) the main bedding material used is straw or similar materials for calves aged under 2 weeks. Poorly managed straw bedding, in particular, provides an ideal breeding ground for coliform bacteria (53). Thus, in order to prevent umbilical infections the provision of clean and dry bedding material is crucial (44).

In this study, each farm was only visited once. Hence, each evaluation represented a mere snapshot of the situation on the farm. In addition, results are based on an average score issued for all lying areas on one farm. These restrictions might explain the lack of significance between the contamination of the lying areas and the omphalitis prevalence, even though the dampness was significant. Future studies should address the impact of the kind of bedding material and its management on umbilical health.

### 4.2 Animal-level factors

#### 4.2.1 Calf

The abdominal hygiene score of the calves had no significant influence on the occurrence of omphalitis. Only 3.9% (*n* = 118) of all calves aged 0–14 days showed moderate or high levels of fecal soiling on their abdominal wall. Because of the small number of soiled calves, it is difficult to determine an association between the abdominal hygiene score and the prevalence of omphalitis. However, it should be noted that this hygiene score is an on-farm assessment that describes the laterally visible soiling of standing calves (18). Compared with the view from the ventral abdomen, the accuracy of these findings can be limited. Moreover, calves are only categorized as “soiled” if dried fecal contaminations are visible on the abdominal wall. Given the good absorption qualities of straw (53), fresh fecal contamination is often missed. However, fresh feces and urine pose a potential risk for pathogens to spread through the umbilical cord into the blood stream of the calf so future studies should aim to incorporate this data.

#### 4.2.2 Dam

An important finding from the present study is the association between the body condition of the dam and the umbilical health of its offspring. Calves from under- or over-conditioned dams were 1.4 times more at risk of omphalitis, respectively, compared to calves from dams in optimum condition. For this analysis calf-dam-pairs were included to describe the association between BCS of the dam after calving and the presence of omphalitis in their offspring on animal level. A limitation of our study is the fact that, as calves between five and 21 days of age were included, their dams were between five and 21 DIM as well. Even though the BCS at 21 DIM is not the same as at calving due to the BCS changes after calving a tendency is ascertainable. The BCS of the dam reflects its health and metabolic status in the dry and transition periods. Dams with a high BCS are at higher risk of dystocia and metabolic disorders post-partum (55), exposing their respective calf to a higher risk of disease. Calves born from dystocia are often less vital. A resulting protracted time until getting up and ingesting colostrum increases the risk for failure of passive transfer (56). To date, the relationship between diseases in the peri-partum period of the dam and the morbidity of the respective calf remains unclear with some authors describing the absence of one altogether (3). Others have found an increased mortality risk of calves from dams with peripartum diseases as mastitis or milk fever, presumably because of a decline in immunoglobulin concentration in its colostrum (57, 58). Although studies about the association between the BCS of the dam and the colostrum immunoglobulin content exist, these findings differ (59) and proving causality poses difficulties (60). Sufficient *in-utero* supply of the calf with nutrients also has a substantial influence on the calf’s development. Over-conditioned or sick cows tend to have a lower feed intake around calving than healthy cows. As a result, the respective calves are more likely to show low birthweights and be more vulnerable to diseases such as scours or respiratory disease (61). In line with this finding, a higher risk for omphalitis has been described in calves born to dams with diseases in the peri-partum period (15).

Overall, the high number of farms visited, the variety of farming approaches and regions represented in the sample as well as the inclusion of related data regarding the dam allowed extensive investigations concerning the influence of factors from different aspects of dairy farming on umbilical health. The large number of data collected, especially animal examinations and evaluation of the housing conditions, forms a solid data set. The findings of this study regarding the high prevalence of omphalitis, the importance of a hygienic environment in the first weeks of life and the importance of dams being in optimum condition around calving correspond with findings from other studies. Nonetheless, there is a lack of research regarding risk factors for omphalitis in dairy calves, especially concerning colostrum management, different ways of navel disinfection (e.g., time after birth, type of application, frequency, type of active substance) and dam’s health.

### 4.3 Limitations

Some limitations of this study were already described. The study was not designed to examine the umbilical health status of all calves on the farms participating, but to create an overview of the health status of dairy herds in Germany by a single farm visit. Thus, neither the medical history nor follow-up examinations of calves were available. Additionally, the classification of “healthy” or “omphalitis” based on one sign of inflammation (swelling with or without warmth, reddening or pain response) poses a few difficulties. Since enlargement of the umbilical region on its own is no reliable symptom for omphalitis (13), false positive diagnoses were possible. Especially in the first few days of life, a swollen umbilicus might not originate from an omphalitis. Moreover, as every calf was only examined once, individual size differences of the umbilicus could not be considered [sex, birth weight, calving difficulties (7)]. Therefore, to reduce the number of false positive cases, calves aged less than 5 days were excluded from this analysis. Furthermore, only the health, hygiene, and husbandry conditions on the day of the farm visit could be considered. It was not possible to rule out certain misjudgments regarding the timing of managerial steps. For instance, on a farm where calf boxes are only re-bedded once a month, a coincidental farm visit on the day after re-bedding of the calf boxes, could have led to skewed nesting scores and assessments of dampness.

A practical limitation of information gathered by questionnaire is that a possible difference between an instruction stated in the interview and the actual execution by the farmer or farm staff cannot be ruled out (62). Moreover, some interviewees could be prone to answer some questions not correctly to avoid criticism of the interviewer (especially questions concerning animal welfare or legal requirements) (39). Furthermore, as the documentation, especially concerning calf management and health, was improvable or not used on many farms, farmers tend to misjudge certain farm aspects (e.g., treatment incidences).

## 5 Conclusion

This study provides insight into the umbilical health of a large number of neonatal calves on dairy farms in three different regions of Germany. Significant differences in calving management, calf health management and hygienic conditions were observed between regions. Multivariable statistical analyses revealed that calves from farms separating calf and dam more than 12 h after birth have a smaller risk for omphalitis compared to farms with immediate separation. On the contrary, calves that are born to dams with a body condition score outside the target range, are more likely to develop omphalitis in the first 21 days of life. Damp bedding in the first 2 weeks of life presents an additional risk factor for omphalitis. Even though many potentially influential factors concerning umbilical health were included in the analyses, only a few statistically significant associations were identified. This highlights the complexity of the influence of different management approaches and environmental conditions on umbilical health. The results of this study provide suggestions for further investigations into the influence of maternal health, colostrum management and the technique of navel disinfection on umbilical health. Possibilities to enhance the awareness for calf health and welfare, especially concerning umbilical health, need to be implemented in the daily routine of farmers and veterinarians.

## Data Availability

The datasets presented in this article are not readily available because the data were acquired through cooperation between different universities. Therefore, any data transfer to interested persons is not allowed without an additional formal contract. Data are available for qualified researchers who sign a contract with the project consortium. This contract will include guarantees of the obligation to maintain data confidentiality in accordance with the provisions of German data protection law. Currently, there exists no data access committee nor another body who could be contacted for the data; a committee will be founded for this purpose. This future committee will consist of the authors as well as members of the related universities. Interested cooperative partners, who are able to sign a contract as described above, may contact: MH, Clinic for Cattle at the University of Veterinary Medicine, Hannover, Bischofsholer Damm 15, 30173 Hannover, Germany, Email: martina.hoedemaker@tiho-hannover.de. Requests to access the datasets should be directed to martina.hoedemaker@tiho-hannover.de.

## Footnotes

^1^ http://www.dagitty.net/
