# Supplementary material for: Risk factors for omphalitis in neonatal dairy calves
Source: Front Vet Sci. 2024 Nov 25;11:1480851. doi: 10.3389/fvets.2024.1480851 (PMC11625811; doi:10.3389/fvets.2024.1480851)
Supplement: Supplementary file 1 [file Data_Sheet_1.docx]

Supplementary Material

Article Title

First Author*, Co-Author, Co-Author

*** Correspondence:** Corresponding Author: [email@uni.edu](mailto:email@uni.edu)

The following Supplementary Figures 1 to 18 show the causal directed acyclic graphs (DAG) (<http://www.dagitty.net/>) used to examine the association between different influence variables and omphalitis in neonatal dairy calves.
Each DAG includes the target variable “Ex_omphalitis” the respective influence variable (green variable) and all possible confounder variables.

The arrows drawn demonstrate the connection between the different variables. The confounder variables (pink variables and arrows) were marked by the program (“dagitty”) based on the arrows drawn.
Explanation of the abbreviation for each source the data was retrieved from:

Q: data retrieved by questionnaire
HIT: data retrieved from the National Traceability and Information System for Animals (“Herkunftssicherungs- und Informationssystem für Tiere”, HIT)
DHI: data retrieved from national milk recording system (DHI)
Ex: data collected by clinical examination during a single farm visit
E: data collected by evaluation of the housing conditions during a single farm visit


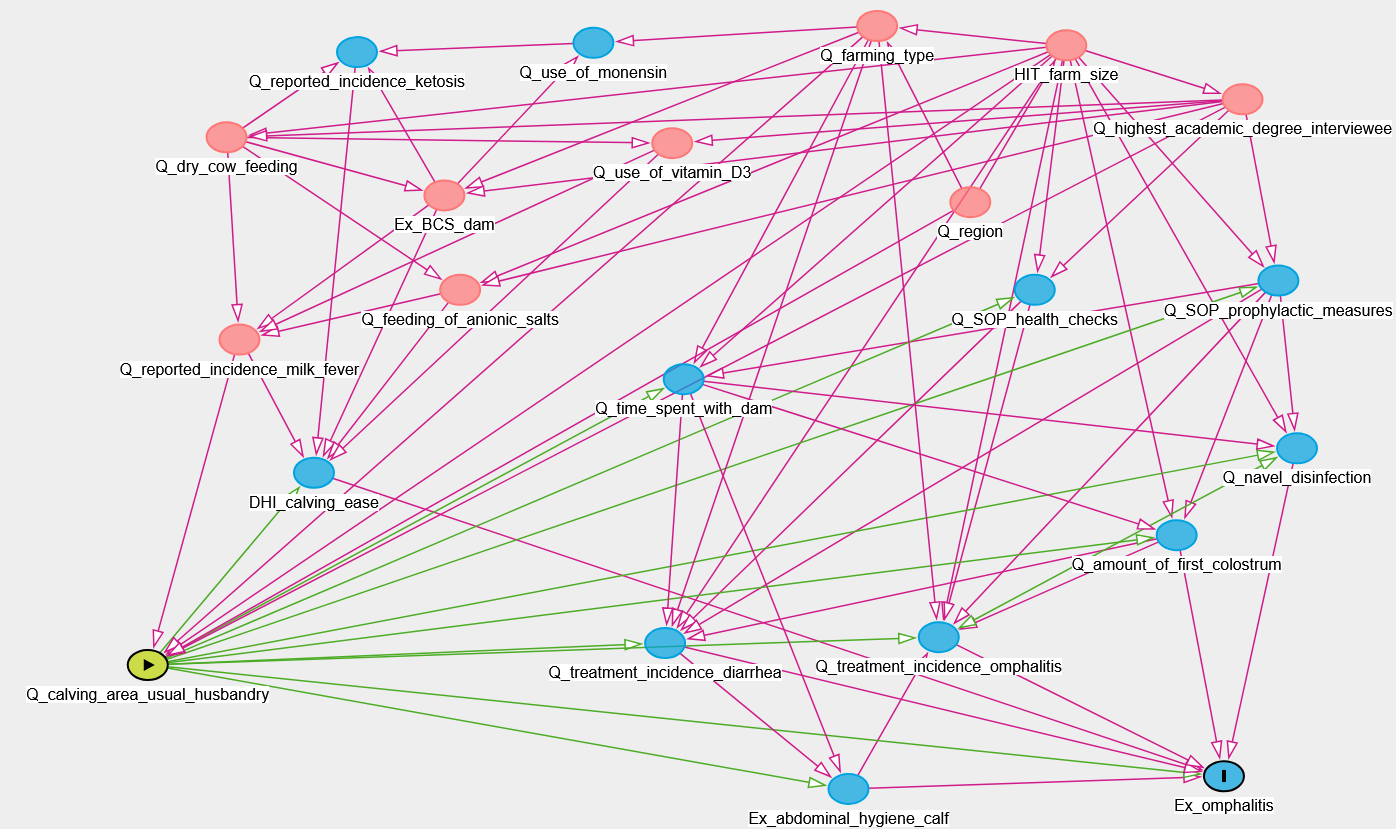


Supplementary Figure 1: Causal directed acyclic graph (DAG) (http://www.dagitty.net/) with "Ex_omphalitis" as target variable, "Q_calving_area_usual_husbandry" as influence variable and all possible confounder variables.
SOP: standard operation protocol
BCS: body condition score
dry cow feeding: single-phase or two-phase feeding of the dry cows


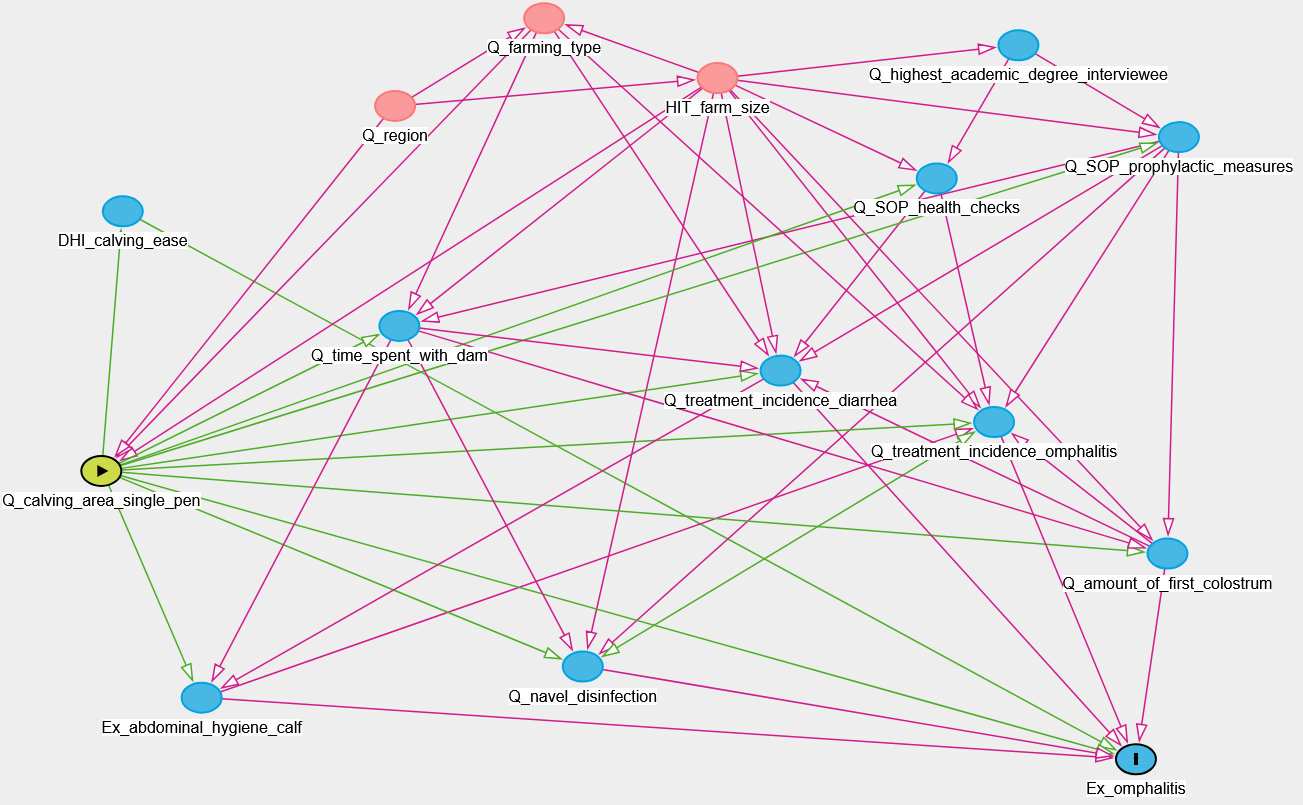


Supplementary Figure 2: Causal directed acyclic graph (DAG) (http://www.dagitty.net/) with "Ex_omphalitis" as target variable, "Q_calving_area_single_pen" as influence variable and all possible confounder variables.
SOP: standard operation protocol


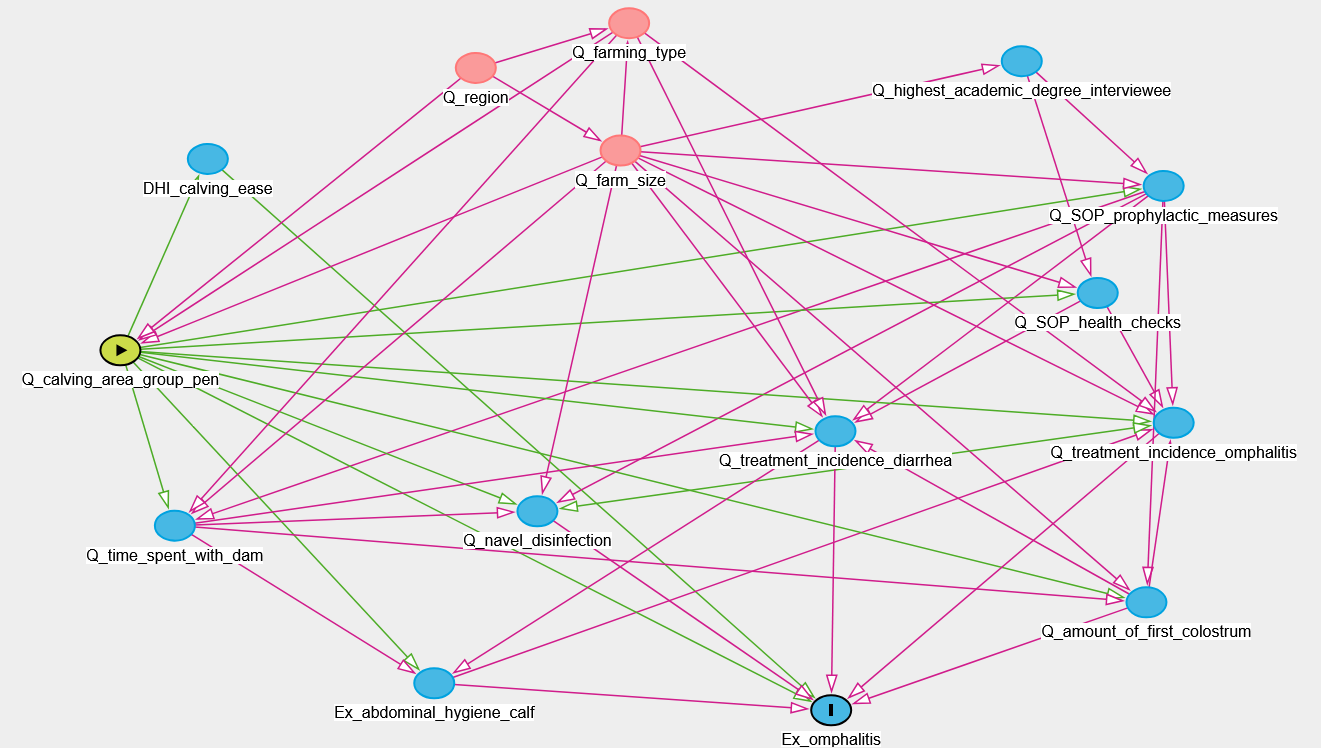


Supplementary Figure 3: Causal directed acyclic graph (DAG) (http://www.dagitty.net/) with "Ex_omphalitis" as target variable, "Q_calving_area_group_pen" as influence variable and all possible confounder variables.
SOP: standard operation protocol


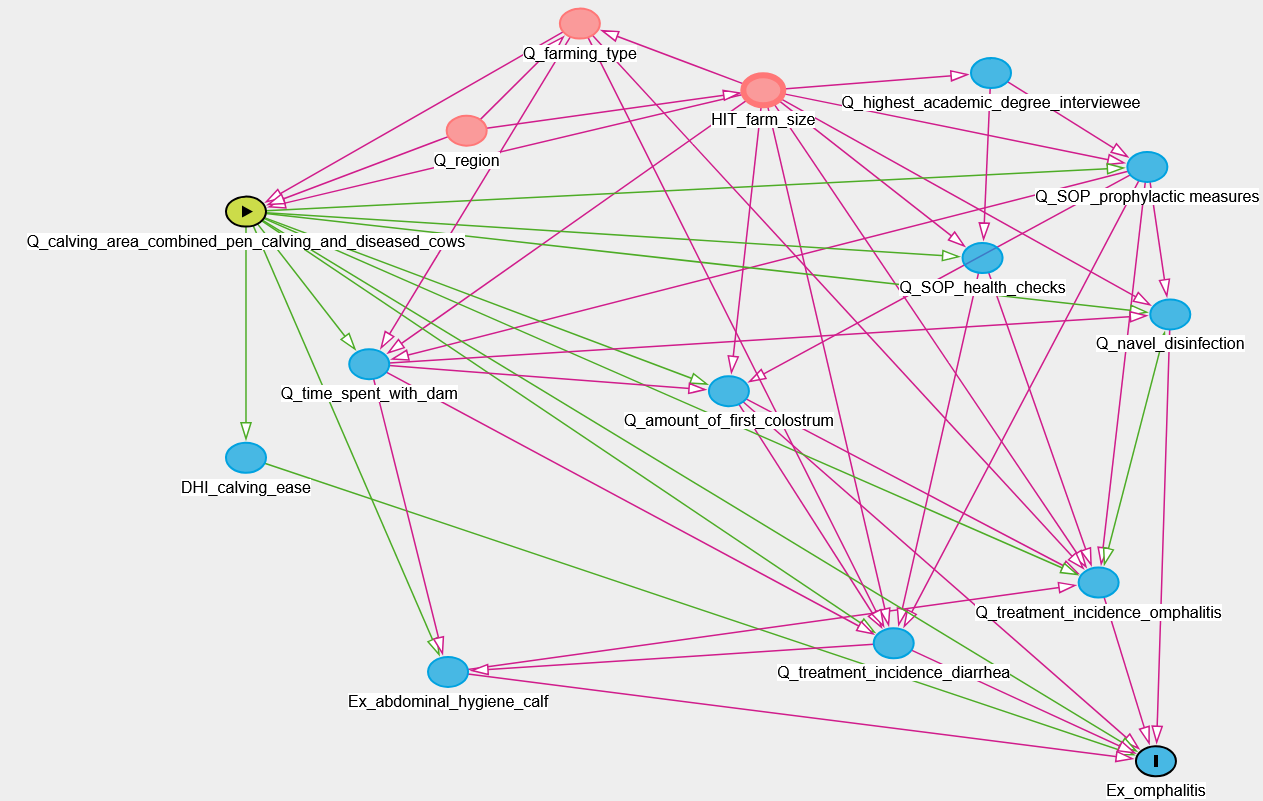


Supplementary Figure 4: Causal directed acyclic graph (DAG) (http://www.dagitty.net/) with "Ex_omphalitis" as target variable, "Q_calving_area_combined_pen_calving_and_diseased_cows" as influence variable and all possible confounder variables.
SOP: standard operation protocol


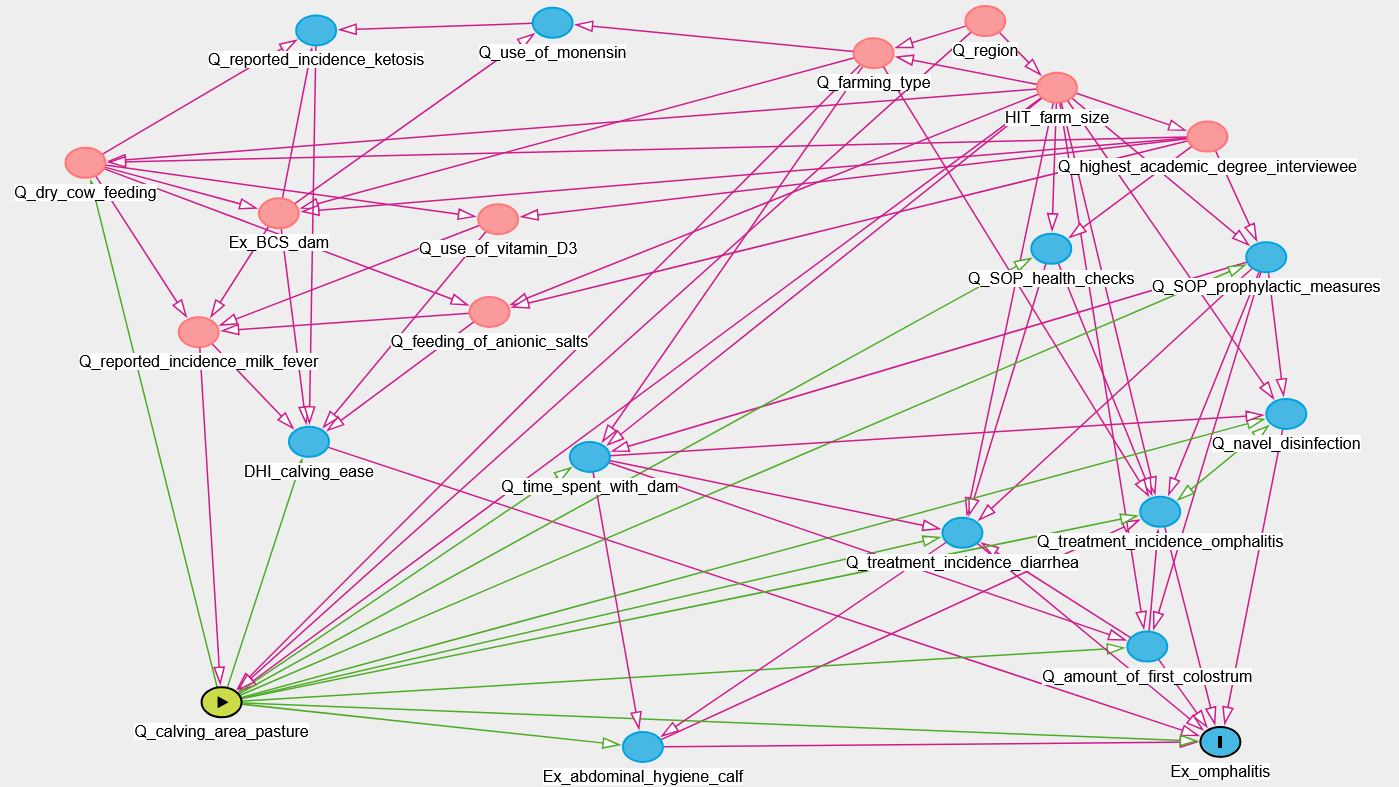


Supplementary Figure 5: Causal directed acyclic graph (DAG) (http://www.dagitty.net/) with "Ex_omphalitis" as target variable, "Q_calving_area_pasture" as influence variable and all possible confounder variables.
SOP: standard operation protocol
BCS: body condition score
dry cow feeding: single-phase or two-phase feeding of the dry cows


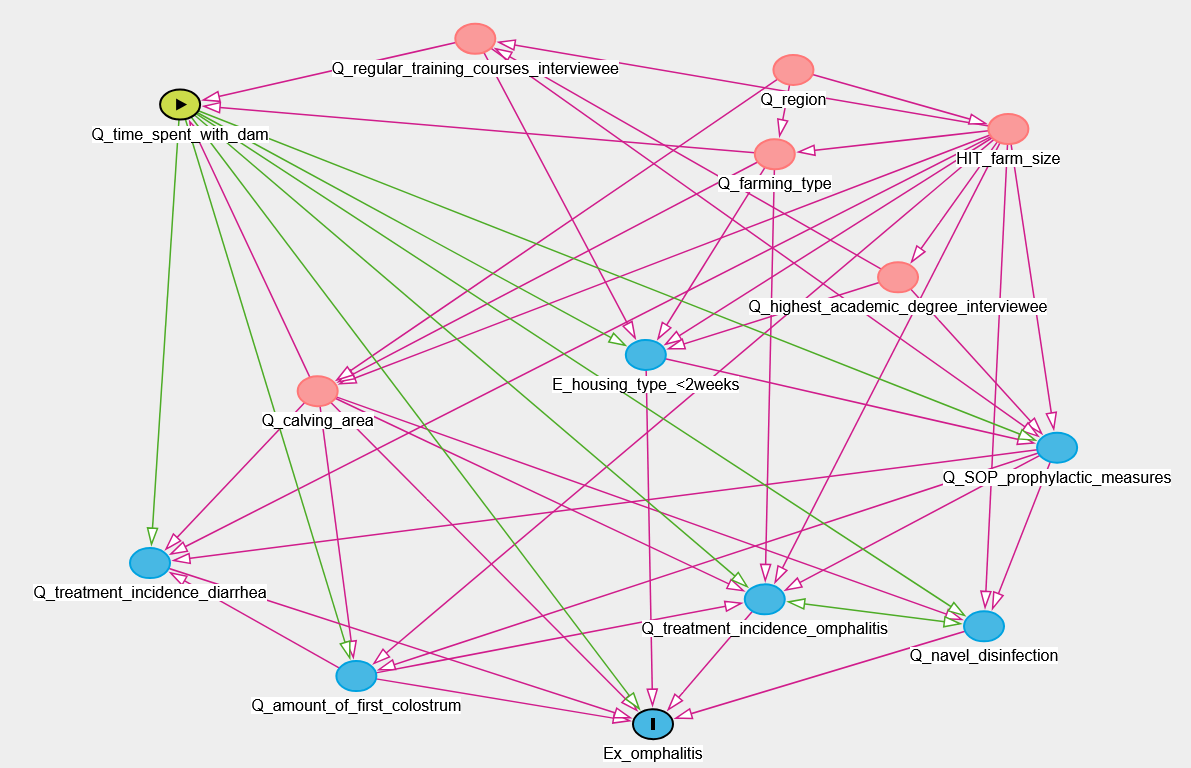


Supplementary Figure 6: Causal directed acyclic graph (DAG) (http://www.dagitty.net/) with "Ex_omphalitis" as target variable, "Q_time_spent_with_dam" as influence variable and all possible confounder variables.
SOP: standard operation protocol


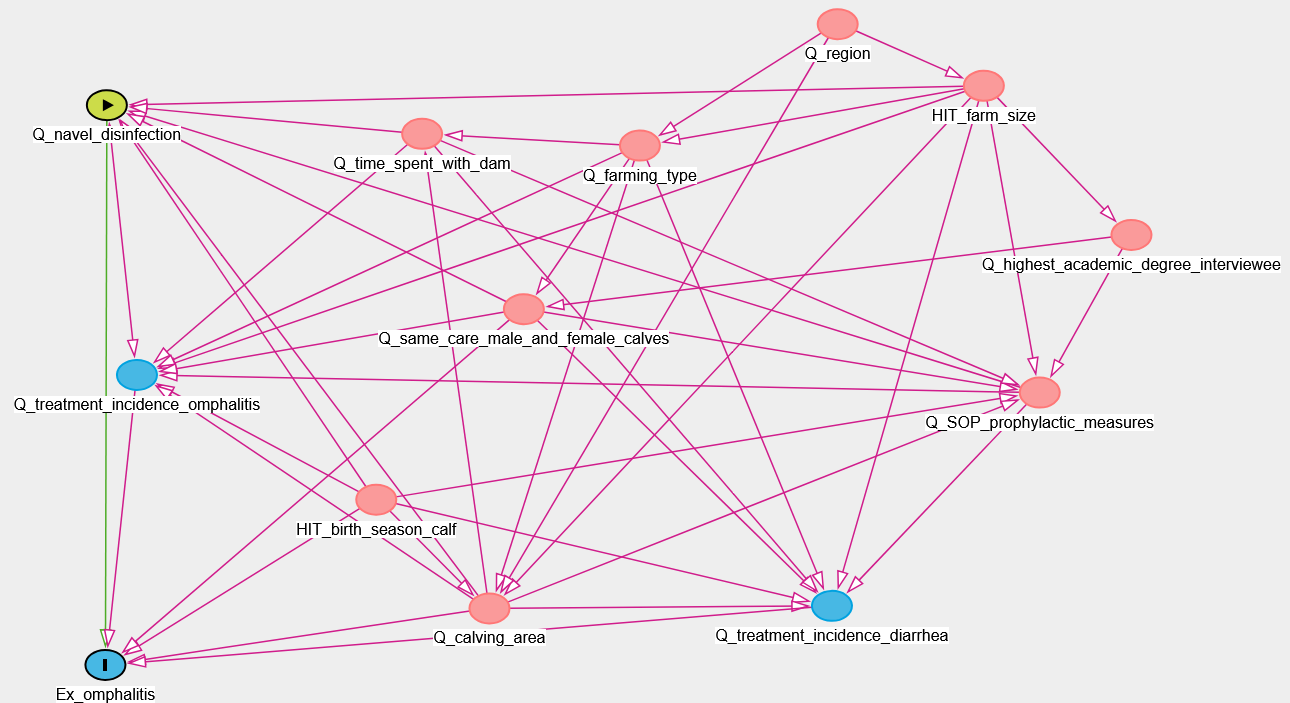


Supplementary Figure 7: Causal directed acyclic graph (DAG) (http://www.dagitty.net/) with "Ex_omphalitis" as target variable, "Q_navel_disinfection" as influence variable and all possible confounder variables.
SOP: standard operation protocol


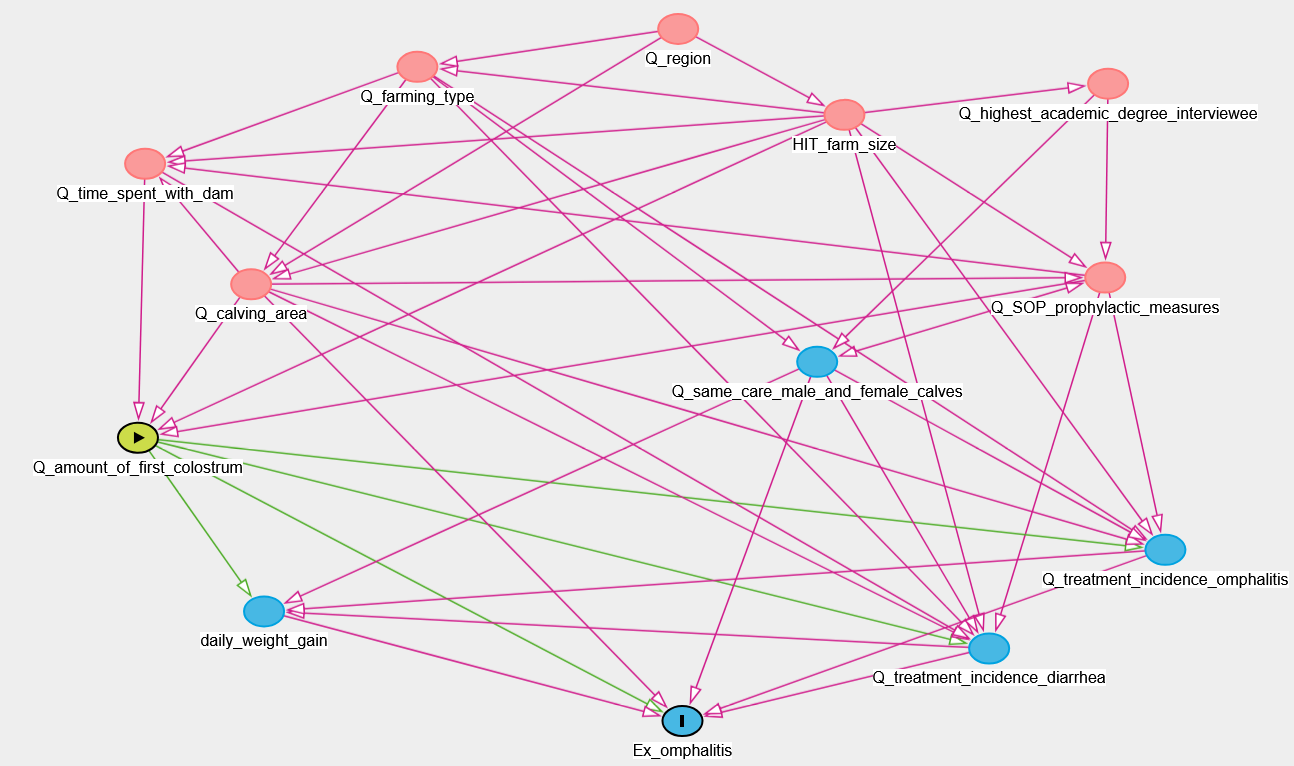


Supplementary Figure 8: Causal directed acyclic graph (DAG) (http://www.dagitty.net/) with "Ex_omphalitis" as target variable, "Q_amount_of_first_colostrum" as influence variable and all possible confounder variables.
SOP: standard operation protocol


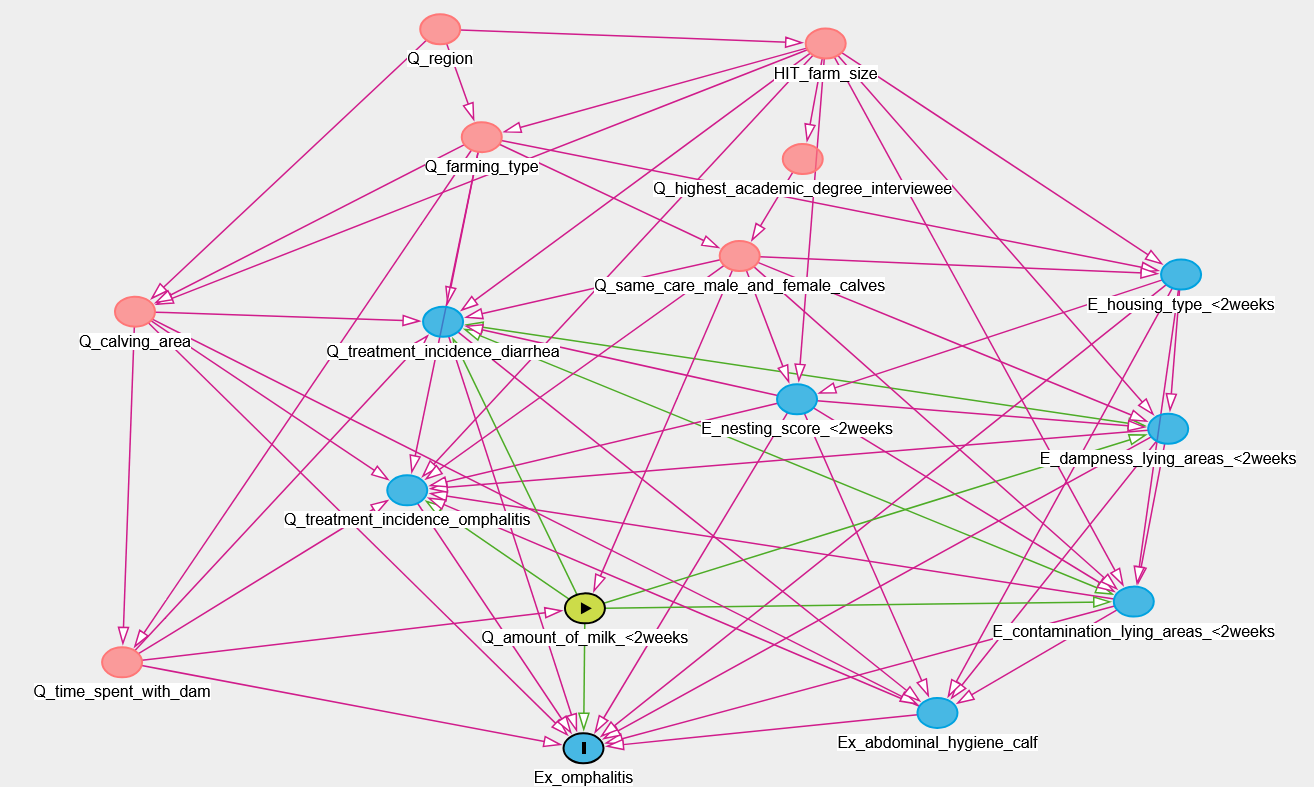


Supplementary Figure 9: Causal directed acyclic graph (DAG) (http://www.dagitty.net/) with "Ex_omphalitis" as target variable, "Q_amount_of_milk_<2weeks" as influence variable and all possible confounder variables.


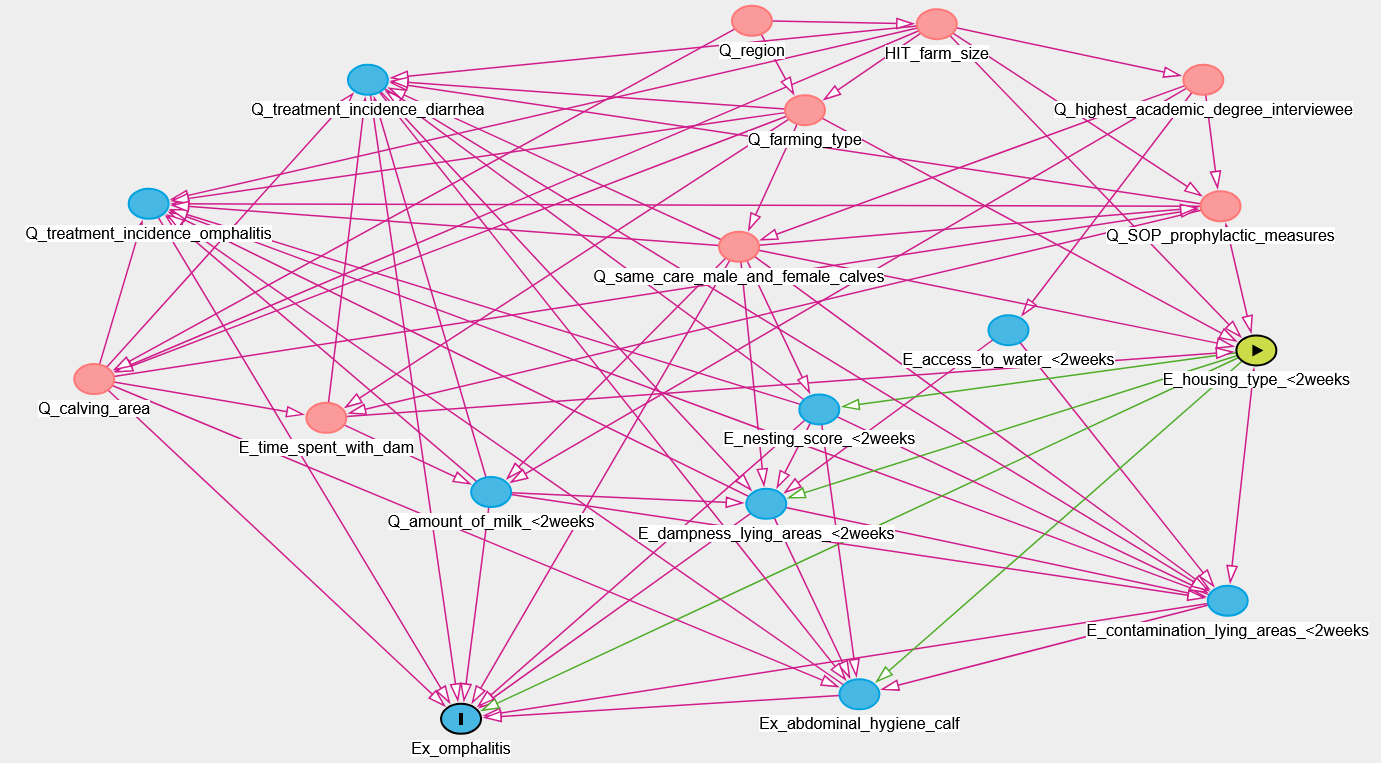


Supplementary Figure 10: Causal directed acyclic graph (DAG) (http://www.dagitty.net/) with "Ex_omphalitis" as target variable, "E_housing_type_<2weeks" as influence variable and all possible confounder variables.
SOP: standard operation protocol


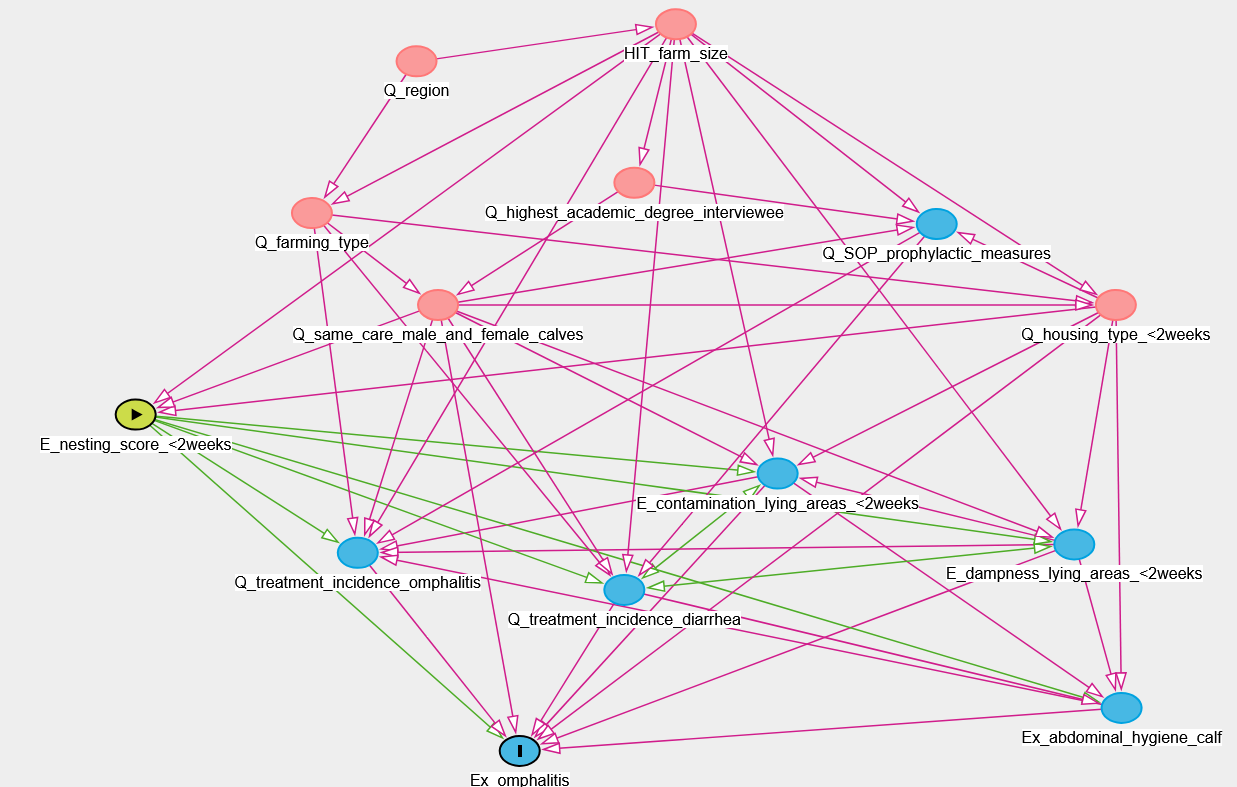


Supplementary Figure 11: Causal directed acyclic graph (DAG) (http://www.dagitty.net/) with "Ex_omphalitis" as target variable, "E_nesting_score_<2weeks" as influence variable and all possible confounder variables.
SOP: standard operation protocol


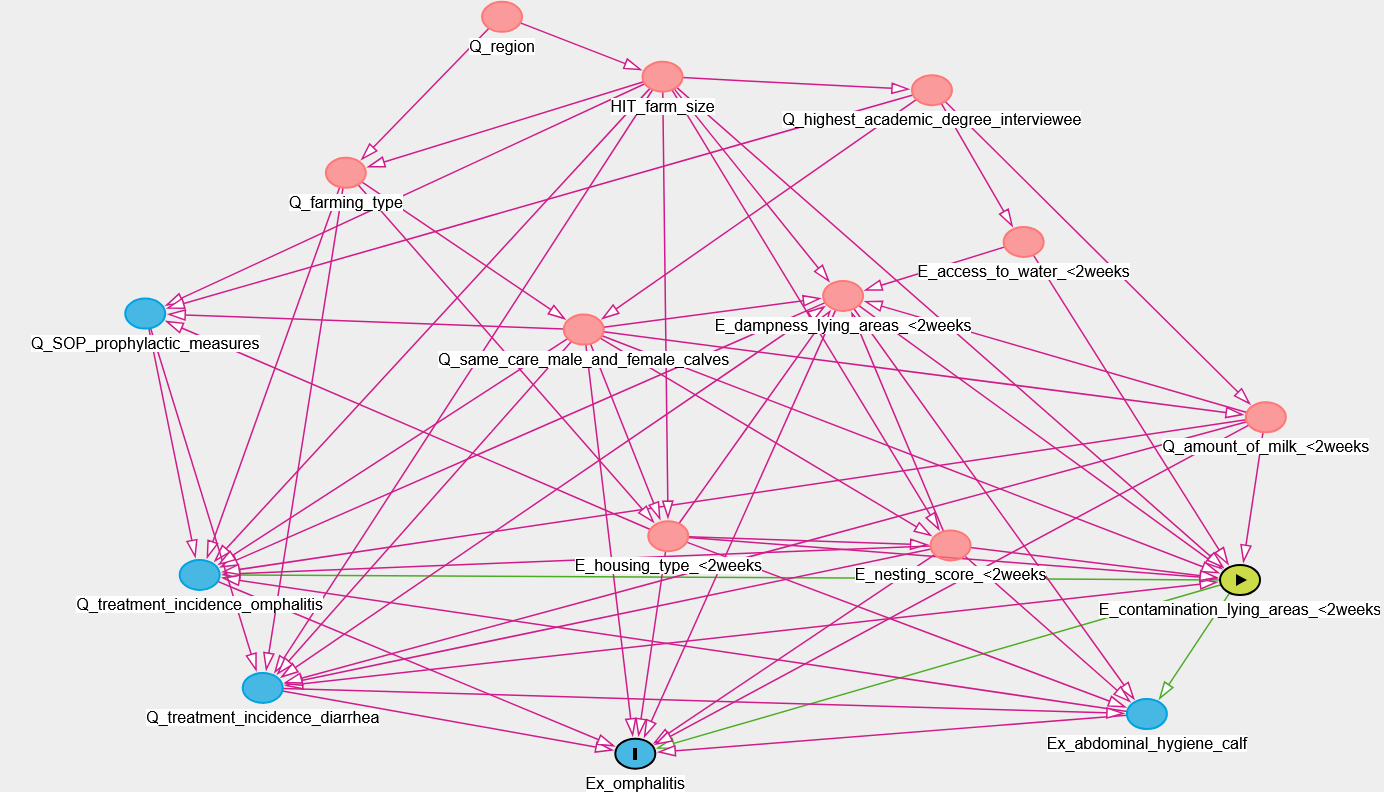


Supplementary Figure 12: Causal directed acyclic graph (DAG) (http://www.dagitty.net/) with "Ex_omphalitis" as target variable, "E_contamination_lying_areas_<2weeks" as influence variable and all possible confounder variables.
SOP: standard operation protocol


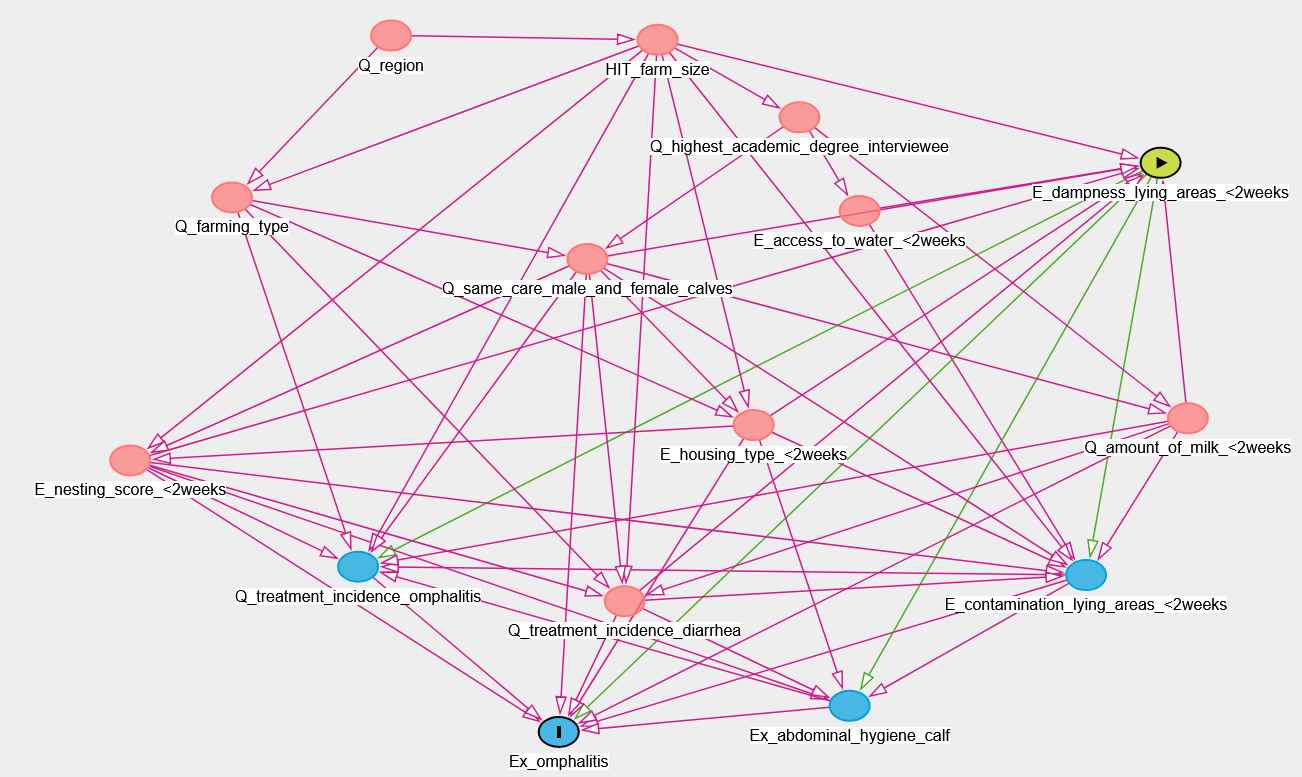


Supplementary Figure 13: Causal directed acyclic graph (DAG) (http://www.dagitty.net/) with "Ex_omphalitis" as target variable, "E_dampness_lying_area_<2weeks" as influence variable and all possible confounder variables.


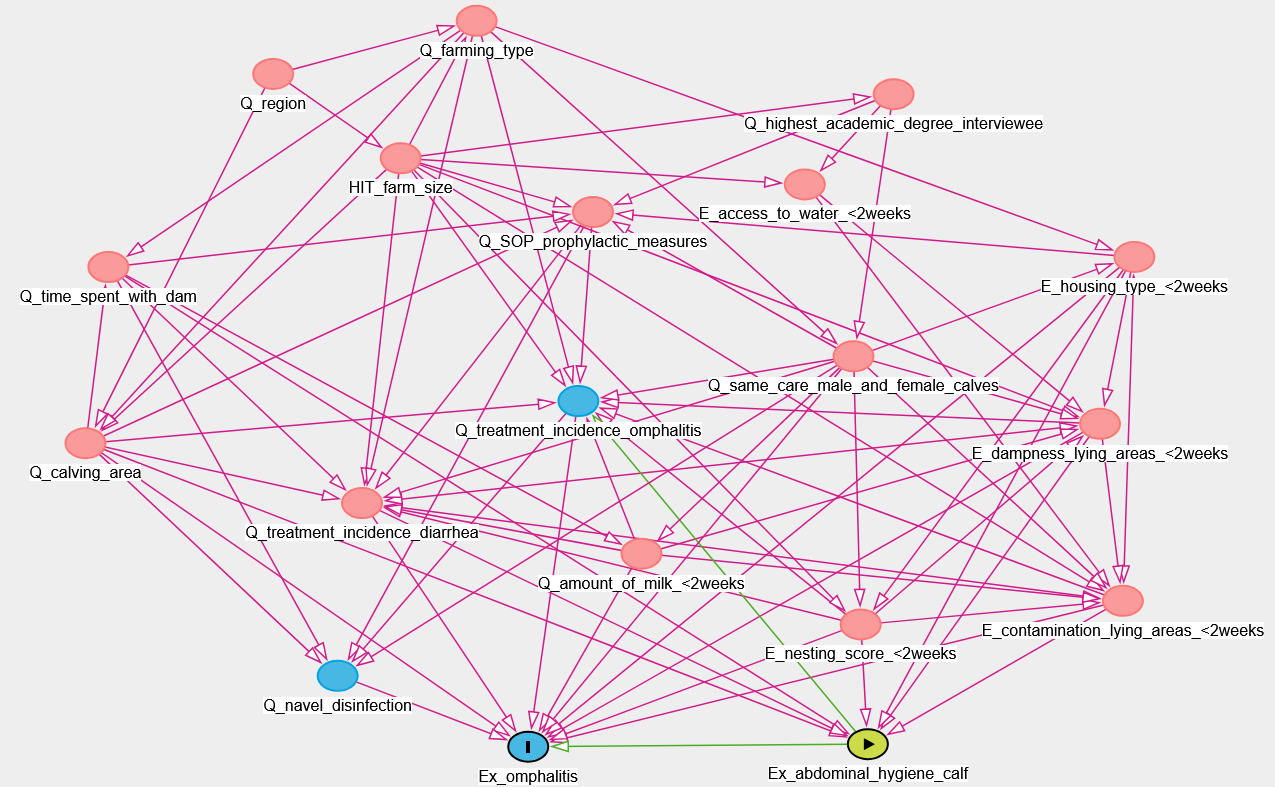


Supplementary Figure 14: Causal directed acyclic graph (DAG) (http://www.dagitty.net/) with "Ex_omphalitis" as target variable, "Ex_abdominal_hygiene_calf" as influence variable and all possible confounder variables.
SOP: standard operation protocol


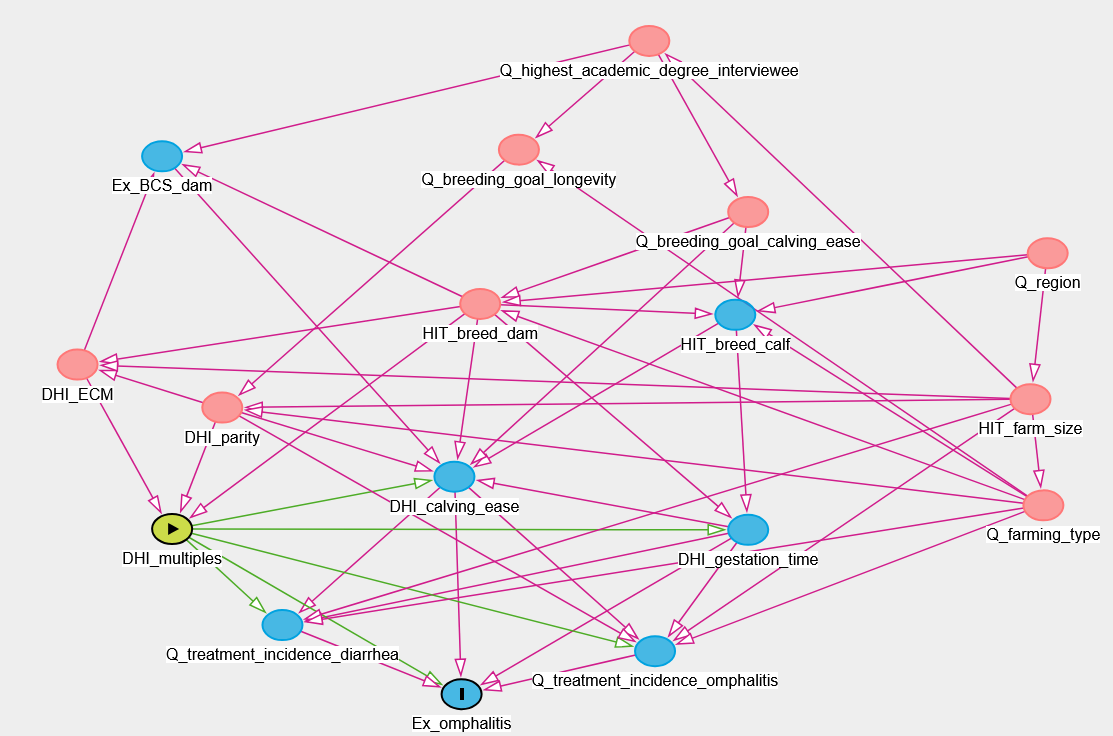


Supplementary Figure 15: Causal directed acyclic graph (DAG) (http://www.dagitty.net/) with "Ex_omphalitis" as target variable, "DHI_multiples" as influence variable and all possible confounder variables.
ECM: Energy Corrected Milk
BCS: body condition score


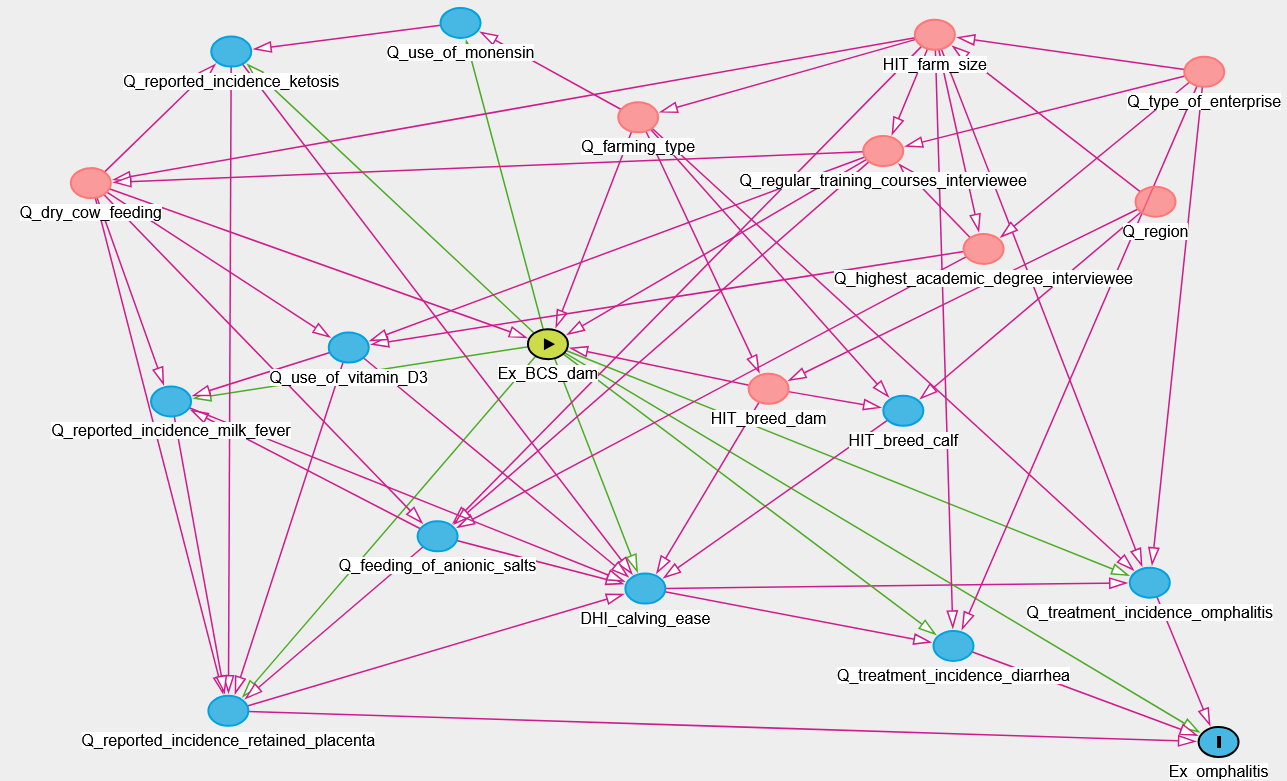


Supplementary Figure 16: Causal directed acyclic graph (DAG) (http://www.dagitty.net/) with "Ex_omphalitis" as target variable, "Ex_BCS_dam" as influence variable and all possible confounder variables.
BCS: body condition score
dry cow feeding: single-phase or two-phase feeding of the dry cows
type of enterprise: dairy farming as main or sideline enterprise


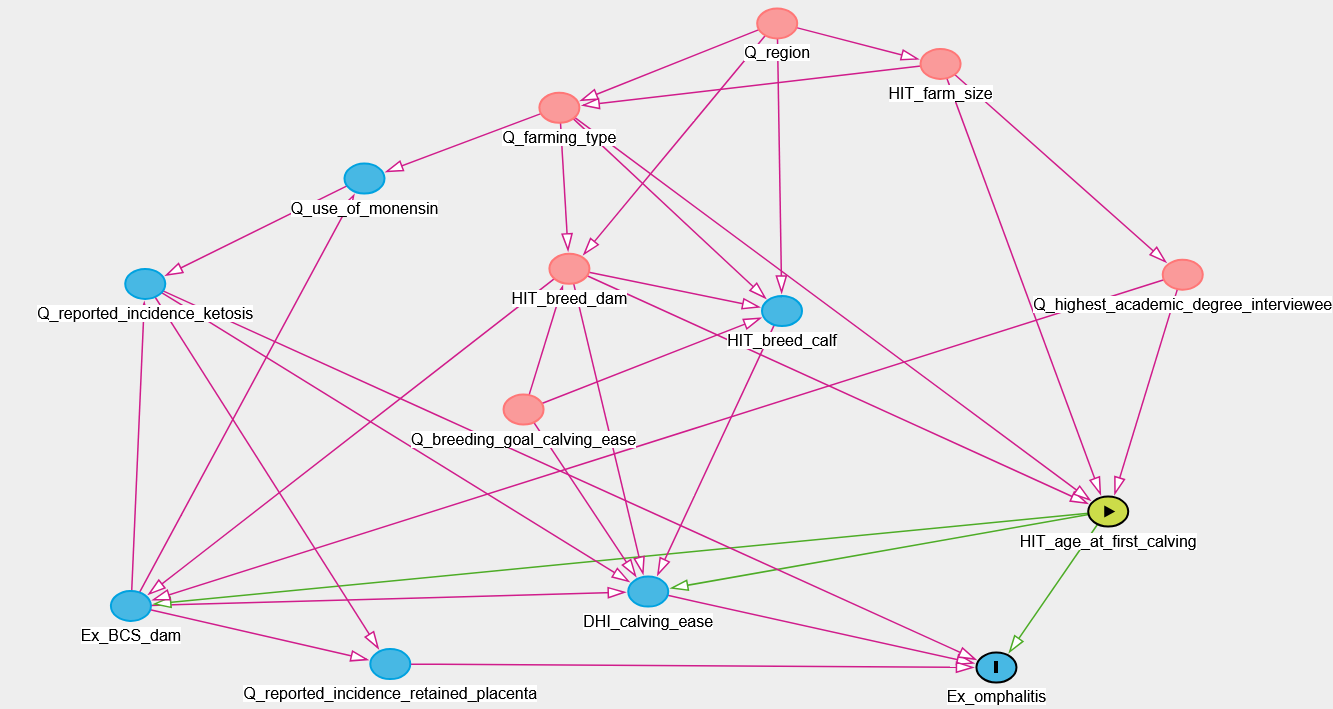


Supplementary Figure 17: Causal directed acyclic graph (DAG) (http://www.dagitty.net/) with "Ex_omphalitis" as target variable, "HIT_age_at_first_calving" as influence variable and all possible confounder variables.
BCS: body condition score


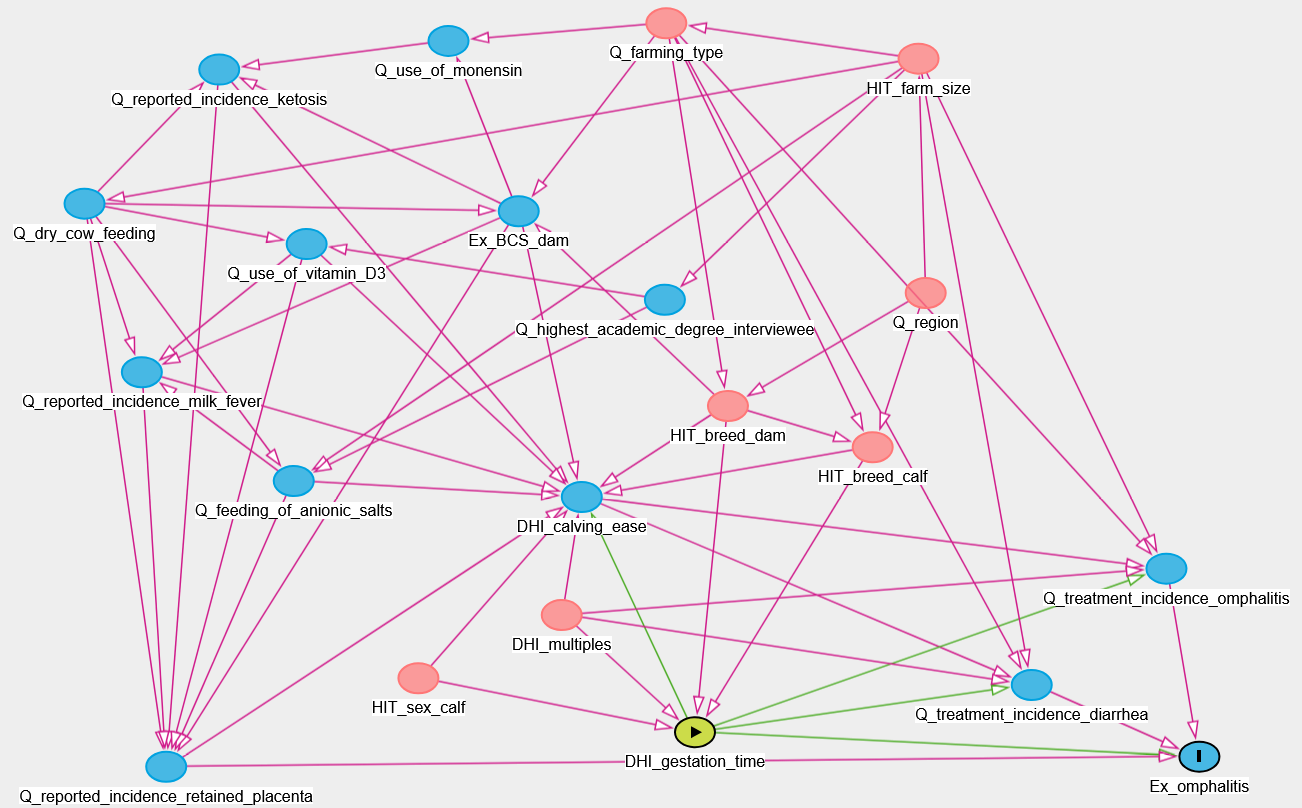


Supplementary Figure 18: Causal directed acyclic graph (DAG) (http://www.dagitty.net/) with "Ex_omphalitis" as target variable, "DHI_gestation_time" as influence variable and all possible confounder variables.
BCS: body condition score
dry cow feeding: single-phase or two-phase feeding of the dry cows

Table S 1: Description of the study population and univariable association of confounder variables with omphalitis in neonatal dairy calves from 567 German dairy farms for all three regions on farm level.

| **Variable** | **Number of farms (%)** | **Crude estimate** | **OR** | **95% CI** | | **p-value** |
| --- | --- | --- | --- | --- | --- | --- |
| **Region** |  |  |  |  |  | **<0.0001** |
| North | 180 (31.7) | Reference | . | . | . | . |
| East | 216 (38.1) | -0.594 | 0.55 | 0.48 | 0.63 | <0.0001 |
| South | 171 (30.2) | -1.138 | 0.32 | 0.25 | 0.41 | <0.0001 |
| **Farming type** |  |  |  |  |  | **0.035** |
| Conventional | 515 (90.8) | Reference | . | . | . | . |
| Organic or in transition | 52 (9.2) | -0.376 | 0.69 | 0.48 | 0.99 | 0.044 |
| **Farm size (log)** | - | -0.01 | 0.99 | 0.92 | 1.06 | **0.776** |
|  |  |  |  |  |  |  |
| **Energy corrected milk yield of the farm** | - | 0.015 | 1.02 | 1.00 | 1.03 | **0.111** |
| OR: odds ratio  95% CI: 95% confidence interval  log: logarithmic scale  IQR: interquartile range | | | | | | |

Table S 2: Description and univariable association of confounder variables with omphalitis in neonatal dairy calves from 567 German dairy farms concerning calves and dams for all three regions on animal level.

| **Variable** | **Number of animals (%)** | **Crude estimate** | **OR** | **95% CI** | | **p-value** |
| --- | --- | --- | --- | --- | --- | --- |
| **CALF** |  |  |  |  |  |  |
| **Breed^1^** |  |  |  |  |  | **0.593** |
| Other dairy breeds and their cross breeds^2^ | 119 (3.5) | Reference | . | . | . | . |
| Holstein^3^ | 2,606 (76.0) | 0.272 | 1.31 | 0.69 | 2.50 | 0.407 |
| Simmental | 451 (13.1) | -0.216 | 0.81 | 0.06 | 10.94 | 0.871 |
| Cross breed between dairy and beef/dual purpose breed with emphasis on beef^4^ | 254 (7.4) | 0.448 | 1.56 | 0.78 | 3.15 | 0.21 |
| **DAM** |  |  |  |  |  |  |
| **Parity** |  |  |  |  |  | **0.003** |
| 1 | 1,064 (32.5) | Reference | . | . | . | . |
| 2 | 847 (25.8) | 0.199 | 1.22 | 1.00 | 1.49 | 0.05 |
| 3 | 566 (17.3) | 0.269 | 1.31 | 1.05 | 1.63 | 0.018 |
| 4 or more | 800 (24.4) | 0.367 | 1.44 | 1.18 | 1.76 | 0.003 |
| **Primipara/multipara^1^** |  |  |  |  |  | **0.003** |
| Primipara | 1,186 (35.5) | Reference | . | . | . | . |
| Multipara | 2,158 (64.5) | -0.238 | 0.79 | 0.67 | 0.92 | 0.003 |
| ^1^ Confounder  ^2^ Brown Swiss, Jersey, German black pied cattle, German red pied cattle, Angler, cross breed   between 2 dairy breeds  ^3^ Black and Red Holstein  ^4^ Cross breeds between dairy and beef breed or 2 beef breeds, Pinzgauer cattle, other  OR: odds ratio  95% CI: 95% confidence interval | | | | | | |
